# “Daily Growth” Parenting Program Delivered via Ecological Momentary Intervention: Pilot Randomized Controlled Trial of a Prototype

**DOI:** 10.2196/87917

**Published:** 2026-09-14

**Authors:** Stefanie Ewald, Tomer Savariego Berkowitz, Storm Hiskens-Ravest, Kelsie Bufton, Maria Bates, Eric O, Matthew Fuller-Tyszkiewicz, Gabriella Louise King, Elizabeth Mary Westrupp

**Affiliations:** 1 School of Psychology, Faculty of Health, Deakin University, 221 Burwood Hwy, Burwood, Victoria, 3125, Australia, +61 0405 708 782; 2 School of Communication and Creative Arts, Faculty of Arts and Education, Deakin University, Burwood, Victoria, Australia; 3 Department of Digital Engagement, Deakin University, Burwood, Victoria, Australia

**Keywords:** Daily Growth, emotion coaching, Active Play, emotion socialization, intervention, parenting, emotion regulation, child development, ecological momentary intervention

## Abstract

**Background:**

Despite evidence showing that parenting interventions reduce rates of child mental health problems, their reach and engagement remain low. Daily Growth is a digital parenting program designed to improve parent and child emotion regulation using an ecological momentary intervention (EMI) approach. Daily Growth sends twice-daily prompts and delivers 3-minute videos tailored to a recent parenting situation, offering 2 program types (Emotion Coaching and Active Play) to meet different parent preferences. EMI has rarely been tested with parenting programs.

**Objective:**

This pilot aimed to assess the feasibility and acceptability of the EMI design and a prototype of Daily Growth, offering the 2 programs via 10 of 60 planned videos.

**Methods:**

A pilot randomized controlled trial recruited 189 Australian parents of children aged 2‐4 years, randomized to either intervention (n=94) or active control (n=95). Participants completed a 25-minute online survey at baseline and at conclusion of the program period. In the intervening 2 weeks, participants received twice-daily 1-minute pre-EMI surveys via email. Participants who reported any parent or child negative affect or emotion dysregulation received a resource. Participants in the intervention condition were randomized in the moment to receive a 3-minute Emotion Coaching or Active Play video, while participants in the control condition received a link to a parenting website. Participants were sent a 1-minute post-EMI survey. For both conditions, parenting support was tailored to 1 of 5 predefined parenting situations.

**Results:**

Overall, 71% (135/189) of participants completed the 2-week post survey, and over two-thirds completed at least half of the daily pre-EMI surveys. Despite technical issues, most participants would recommend Daily Growth to other parents (53/67, 79%), use the skills learned (46/67, 69%), reported it as easy to use (45/67, 67%), and were satisfied with Daily Growth (40/67, 60%).

**Conclusions:**

This pilot evaluated whether brief EMI resources could engage parents in real time. Findings suggest that the Daily Growth prototype was feasible and well received, supporting further implementation in a larger trial.

## Introduction

### Background

Emotion regulation skills influence all aspects of human functioning and are central in the etiology of mental health disorders [1,2]. Parents play a vital role in the development of their child’s emotion regulation skills [3,4], and both parenting beliefs and practices are modifiable. While parenting interventions demonstrate strong effectiveness in improving child emotion regulation and reducing child mental health problems [5,6], their population reach has been extremely low (<10% population) [7,8]. This limited reach disproportionately affects fathers, single parents, and Aboriginal and Torres Strait Islander, migrant, culturally and linguistically diverse, and rural or remote families [8-11]. In response, several parenting interventions have transitioned to online delivery formats to increase access. More recently, smartphone-based parenting apps have further expanded access [12-17]. Online and app-based programs have the potential to make support more accessible, cost-efficient, and anonymous, and have been welcomed by users [13-16,18-20]. However, despite demonstrated efficacy, digital parenting interventions continue to face hurdles such as low uptake, engagement, and adherence [21-24], suggesting that online delivery alone does not fully address barriers to uptake and engagement. At the same time, many parenting programs focus on child behavior change rather than emotion regulation [25-27] and are targeted toward school-aged children or adolescents, despite early childhood representing a critical time for children’s emotional development [28]. Together, these limitations point to a need for parenting programs that address both how support is delivered (moving beyond access to sustained engagement) and what is delivered, with a focus on emotion regulation in early childhood.

### This Study: Daily Growth

This study is part of a larger research project developing and testing Daily Growth, a personalized, stand-alone parenting app for parents of children aged 2‐4 years. Daily Growth aims to enhance parent emotion regulation and emotion-related parenting skills, ultimately supporting children’s emotion regulation and reducing rates of child mental illness. To address barriers to reach, access, and engagement in population-level parenting programs, the platform integrates novel technological approaches successfully applied in adult health settings [29-32]. In collaboration with parents, carers, and community practitioners, we co-designed Daily Growth using the Design Mapping framework to deliver tailored, in-the-moment parenting support using an ecological momentary intervention (EMI) design. The program offers microinterventions (brief 3-minute video resources) matched to specific parenting situations and combines 2 evidence-based parenting approaches: Emotion Coaching and Active Play.

EMIs deliver brief, frequent support via smartphone apps, aiming to seamlessly integrate into daily life [33,34]. In adult mental health settings, EMI approaches have demonstrated improvements in adherence and reductions in mental health symptoms (eg, gambling, stress, anxiety, and body satisfaction), while requiring less time and fewer resources than traditional interventions [31,32,35,36]. Despite this promise, EMI designs have rarely been tested within parenting interventions [13,37,38]. Of the existing studies, one targeted homeless adolescent mothers (n=49) and demonstrated high acceptability and sustained engagement, with participants reporting that the technology supported parent emotion regulation during stressful parenting moments [13]. A second study was a small feasibility study (n=33) exploring daily reflective questions delivered via ecological momentary assessment (EMA) to increase parental awareness of child emotions and support in-the-moment emotion coaching, suggesting that parents can sustain engagement with daily prompts, and that doing so can increase their attention to child emotions in everyday life [37]. A third study has proposed, but not yet tested, an EMI-based mindfulness intervention for parents of children with autism spectrum disorder (n=670) [38]. No studies to date have examined EMI-based parenting programs that are population-based, target the early childhood period, or specifically aim to build children’s emotion regulation skills, and it remains unclear whether this approach will be feasible, engaging, and relevant for parents navigating busy daily routines. A further limitation of previous parenting interventions has been their one-size-fits-all approach, which does not align with evidence that parents vary in the situations and approaches that may be most relevant [20,39-41] and beneficial to them [42]. Tailoring brief microinterventions to parents’ needs may increase the relevance of parenting support and encourage sustained engagement over time. Additionally, collaborating with end users in mental health research has been shown to improve program relevance, engagement, and effectiveness [43]. Integrating tailoring with a co-design approach may therefore strengthen both the relevance and usability of digital parenting support.

Daily Growth was developed according to these principles, delivering brief, tailored resources informed by input from parent end users and professionals. It provides in-the-moment support through 2 distinct programs, recognizing that parents support child emotion regulation through multiple pathways. Emotion Coaching is based on Gottman et al.’s 5 steps [44,45]: noticing emotions, viewing them as teaching opportunities, responding with empathy, labeling emotions, and problem solving. Daily Growth adds a sixth step (reflect), encouraging parents to pause when calm, consider their responses, and model repair. Active Play offers a nonverbal, embodied approach suited to parents less comfortable with emotional talk. Drawing on evidence that physical activity and imaginative play support mood regulation, calm the nervous system, and strengthen executive function tied to emotion regulation [46-48], the program provides practical play-based activities to support parent-child connection and emotion regulation.

This pilot study evaluates the feasibility and acceptability of the prototype version of Daily Growth and its EMI delivery approach, delivered over 2 weeks, compared with an active control condition. Specifically, we aimed to assess:

1. The feasibility of the prototype version of Daily Growth in terms of parent recruitment rates, retention, and flow through all stages of the study, as well as parent engagement via completion rates of the baseline survey, 2-week post survey, and daily prompts.
2. The acceptability of the 10 prototype video resources and the program delivery method via twice-daily pre- and post-EMI prompts.
3. The descriptive efficacy of the prototype version of the Daily Growth program in improving parent and child emotion regulation, mental health, and emotion coaching parenting behaviors in the moment and postintervention compared with the control condition.

We anticipate that predefined feasibility benchmarks will be met, including meeting the target of recruiting at least 150 participants, attrition below 30%, and average completion of at least 50% of pre- and post-EMI prompts. This engagement rate is informed by rates reported in parenting EMI and EMA studies, in which 70% of parents engaged with at least 80% of daily EMA prompts in a general parenting population [37] and approximately 40% of prompts were completed among a vulnerable population of homeless adolescent mothers [13]. Acceptability is considered high if 70% of intervention participants indicate that they are satisfied with the program, informed by satisfaction rates reported in parenting EMA or EMI studies [13,37]. As this is a pilot trial, the primary focus is on feasibility and acceptability rather than efficacy testing. Descriptive outcome data are reported to assess the feasibility of the outcome measurement protocol, including the sensitivity of measures to detect change and the completeness of data collection, in preparation for the definitive trial [49,50].

## Methods

### Research Design

**Figure 1.**
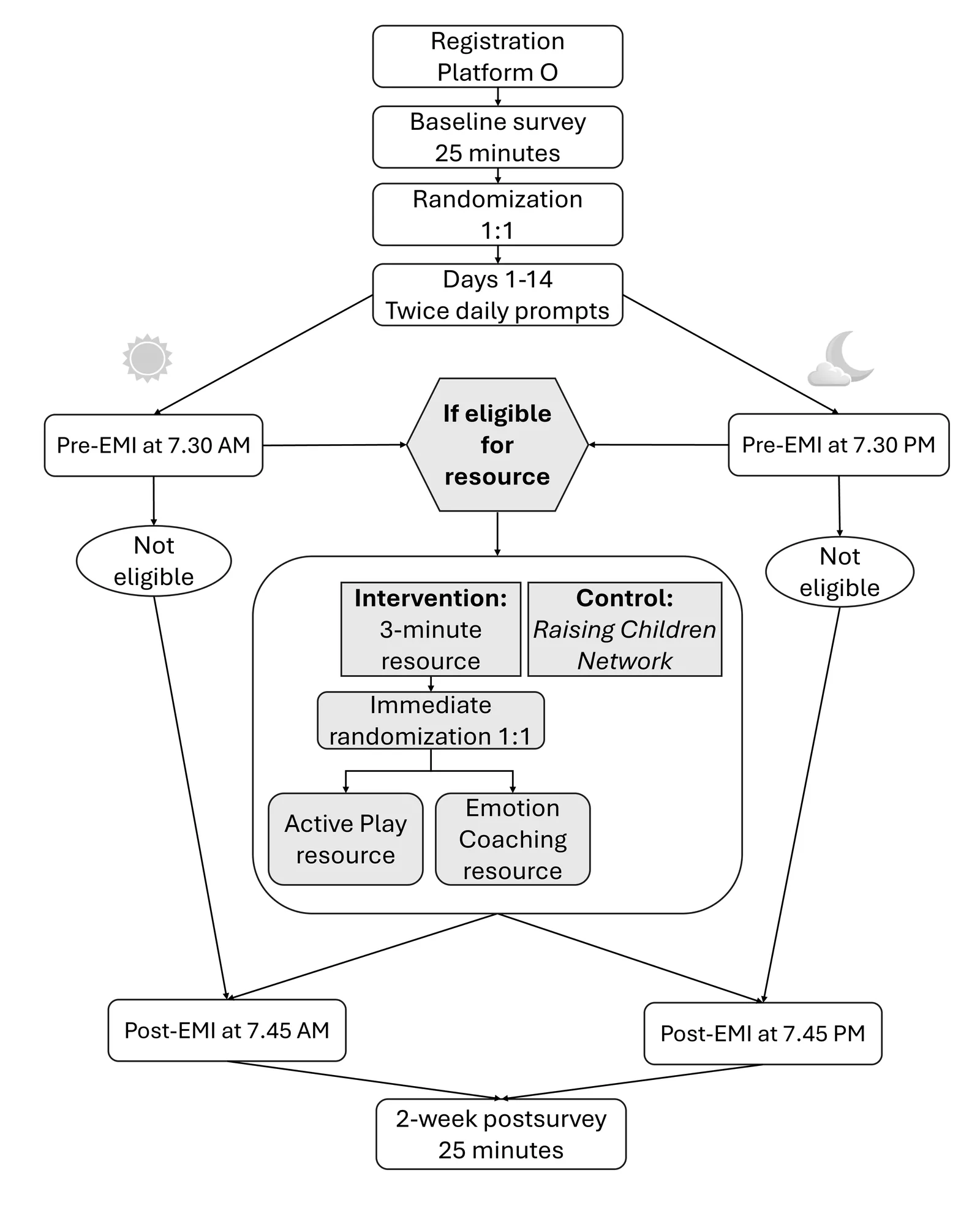
Overview of trial design and ecological momentary intervention (EMI) schedule. Pre-post–EMI=brief,<1-minute survey before or after ecological momentary intervention. Platform O=Platform delivering the Daily Growth prototype. EMI: ecological momentary intervention.

The study design is demonstrated in Figure 1. This study used a pilot randomized controlled trial design with a 1:1 allocation ratio to evaluate the feasibility, acceptability, and descriptive efficacy of a prototype version of the Daily Growth parenting program. The trial was conducted fully online. Participants completed baseline measures via Qualtrics (Qualtrics LLC), followed by a 2-week EMI period during which they received twice-daily pre- and-post-EMI prompts delivered via email. Participants completed a 2-week postprogram survey at the conclusion of the EMI period.

### Ethical Considerations

Study procedures were approved by the Deakin University Human Ethics Advisory Group (HEAG-H 188_2022). Participants provided informed consent online via a checkbox before participation to indicate their consent to participate and be contacted about future research. Participants received a Plain Language Statement outlining the study procedures and purpose, estimated duration of each study component, their right to withdraw at any time, the policy regarding data storage, and contact details for the principal investigator for questions or concerns.

### Participants and Recruitment

Eligibility criteria included residing in Australia, aged 18 years and older, parent or carer of a child aged 2‐4 years, English proficiency, internet access, and basic digital literacy (able to receive emails and complete online surveys). Recruitment methods included contacting parents from a registry of participants from related studies who had previously consented to be contacted for future research, posting in Facebook parenting groups, and running paid Facebook and Instagram advertisements. Parents were reimbursed with Coles gift vouchers based on EMI survey completion rates (50%‐69%=AU$20 (AU$1=US $0.67 as of March 18, 2023); 70%‐84%=AU$30; and 85%‐100%=AU$50). No formal power calculation was conducted as the pilot aimed to test feasibility rather than efficacy. A target of 150 participants was set to ensure sufficient data for feasibility assessment. The final sample comprised 189 parents and caregivers (94 intervention, 95 control).

### Procedure

Participants registered on the online platform “Platform O” and completed a baseline survey via Qualtrics (Qualtrics LLC), an online survey platform. The baseline survey comprised 224 items, taking approximately 25 minutes to complete. The purpose of the questionnaire was to collect demographic information and to assess parent and child emotion regulation, mental health, and emotion coaching parenting practices. No completeness checks were included; however, participants who did not complete the demographics section were removed from the dataset. An attention check was included, and respondents could not review or change their answers. The research team regularly monitored survey responses for suspicious submissions (eg, duplicate entries, bot responses, and participants located outside of Australia) and excluded illegitimate participants. Following completion of the baseline survey, participants were randomly assigned to either the control or intervention condition on a 1:1 basis using Qualtrics’ “Randomiser” feature on completion of the baseline survey. Participants were blinded to their allocation but research team members were not. Participants received their first EMI survey, assessing momentary parent and child affect and emotion regulation, the day after completing the baseline survey. Over the subsequent 14 days, participants received twice-daily email prompts to complete a 1-minute pre-EMI survey at 7:30 AM and 7:30 PM, followed by a 1-minute post-EMI survey prompt at 7:45 AM or 7:45 PM. At the end of the 2-week program period, participants were invited to complete a 2-week post survey via Qualtrics.

### Daily Growth Program and Control Condition

Daily Growth was developed using an iterative co-design process involving parents and professionals. Guided by a novel Design Mapping framework [51], development was informed by (1) an analysis of online parenting discussions to identify common parenting challenges [52], (2) qualitative interviews with parents to understand current use of active play as a parenting strategy [53], (3) structured workshops conducted to co-design program content and delivery [54], (4) ongoing consultation with an advisory group consisting of parents and professionals, and (5) a technical EMA pilot to optimize the timing of prompts [55]. The present pilot evaluated a subset of the full Daily Growth program to assess the feasibility, acceptability, and descriptive efficacy of the program prior to development of the final 6-week smartphone app.

Participants allocated to the intervention condition received access to a prototype version of Daily Growth delivered via the online platform Platform O. Daily Growth is a parenting program designed for parents of children aged 2‐4 years, delivering brief microinterventions using an EMI approach. The prototype included 10 brief 3-minute video resources (5 Emotion Coaching and 5 Active Play) tailored to 5 different parenting situations (mealtime, bedtime, getting dressed, screen time, and sibling conflict). Across the 14-day intervention period, all participants received twice-daily prompts linking to a 1-minute pre-EMI survey. Participants who reported parent or child negative emotion or emotion dysregulation were eligible to receive a tailored parenting resource. Eligible participants selected 1 of 5 difficult parenting situations they had recently experienced or were interested in learning about. Participants in the intervention group were then randomized in the moment to receive either an Emotion Coaching or Active Play video resource tailored to their selected situation. Following resource delivery, participants were prompted to complete a post-EMI survey assessing momentary parent and child affect and emotion regulation. Participants allocated to the active control condition were instead provided with a link to the Raising Children Network, an Australian government–supported parenting information website. The Raising Children Network provides high-quality, but general, parenting information and does not include brief tailored intervention strategies comparable with those delivered by the Daily Growth video resources. The full Daily Growth program currently in development will include 60 video resources delivered over 6 weeks via a custom-built smartphone app for iOS and Android, with twice-daily pre-EMI survey prompts delivered via app notifications [56].

### Outcomes

#### Feasibility

Feasibility was assessed through recruitment, retention, and engagement measures. Recruitment was evaluated based on the ability to achieve the target sample size within the recruitment period. Enrolled participants were defined as those who completed at least the demographic portion of the baseline survey. We aimed for equal representation across child age groups and ≥25% fathers. Retention was measured by attrition rates between baseline and 2-week post surveys. We aimed for <30% attrition in the intervention condition, a benchmark informed by previous intervention studies [11,20,57-61]. Engagement was measured based on the proportion of completed prompts per participant and parent-reported barriers to participation. While EMA studies in adult mental health report close to an 80% average engagement [30,62], Daily Growth reflects the unique context of parenthood, providing flexible, light-touch support without being onerous or interrupting in-the-moment parenting. We therefore prioritized sustained participation over high response rates, targeting 50% pre-EMI completion (1 daily survey on average, providing support 2‐5 times weekly) and 50% post-EMI completion. Engagement and attrition were analyzed by socioeconomic status, gender, and migrant status, as these demographic variables have been associated with differential access to and engagement with parenting programs [9].

#### Acceptability

A 25-item user satisfaction measure combined 5-point scales (eg, “The Daily Growth program meets my needs”), multiple-choice questions (eg, “Which parenting resource did you prefer?”), and open-ended questions (eg, “What problems did you encounter while using the program?"). Items assessed content quality, resource design, research experience, perceived effectiveness, and overall program acceptability. The measure was administered at the end of the 2-week program as part of the 2-week post survey. Acceptability was considered high if the majority (>50%) of intervention participants found the program resources and design satisfactory.

#### Baseline and 2-Week Posturvey Outcome Measures

Study measures are presented in Table 1. The research team selected measures routinely used in large-scale population studies with evidence of good validity and reliability for assessing parent and child emotional and social functioning. The baseline and 2-week post survey collected demographic information and assessed parent and child emotion regulation, mental health, and emotion coaching parenting practices.

**Table 1.** Descriptive statistics (Cronbach α; mean, SD) for baseline and 2-week post survey measures.

Construct	Measure	Subscale	Scale	Items	Baseline			Post survey		
					Values, n	α value	Mean (SD)	Values, n	α value	Mean (SD)
Child measures										
Behavioral problems	MAP-DB^a^ [63]	Temper loss	6	6	189	.91	2.01 (0.92)	135	.90	1.96 (0.87)
Behavioral problems	MAP-DB [63]	Noncompliance	6	4	189	.89	2.13 (1.00)	135	.88	2.17 (1.01)
Behavioral problems	MAP-DB [63]	Aggression	6	5	189	.88	1.10 (0.92)	135	.89	1.07 (0.92)
Depression	SMFQ^b^ [64]	N/A^c^	3	13	189	.73	2.90 (2.61)	133	.79	3.17 (3.04)
Anxiety	SCAS^d^ [65]	GAD and sep anxiety^e^	4	4	189	.71	3.16 (1.90)	134	.73	3.22 (1.93)
Emotion regulation	EERBQ^f^ [66]	Mindfulness	7	2	189	.72	4.01 (1.56)	134	.71	4.56 (1.44)
Emotion regulation	EERBQ [66]	Verbal help-seeking	7	3	189	.71	3.38 (1.43)	134	.78	3.91 (1.57)
Emotion regulation	EERBQ [66]	Verbal venting	7	3	189	.74	3.81 (1.45)	134	.69	4.11 (1.33)
Emotion regulation	EERBQ [66]	Physical venting	7	3	189	.78	2.79 (1.41)	134	.78	2.95 (1.45)
Emotion regulation	EERBQ [66]	Emotion reactivity	7	6	189	.75	4.01 (1.00)	134	.73	3.91 (0.98)
Negative affect	PANAS-C-P^g^ [67]	Negative affect	5	5	189	.82	8.84 (3.09)	133	.77	9.09 (2.89)
Parent measures										
Emotion regulation	DERS^h^ [68]	Total	4	19	189	.94	38.23 (13.92)	133	.94	38.00 (13.35)
Emotion regulation	DERS [68]	Nonacceptance^i^	4	3	189	.85	6.25 (3.01)	133	.87	6.28 (3.11)
Emotion regulation	DERS [68]	Goals	4	3	189	.88	7.51 (3.11)	133	.86	7.47 (2.88)
Emotion regulation	DERS [68]	Impulse control difficulties	4	6	189	.88	11.25 (4.71)	133	.88	11.46 (4.52)
Emotion regulation	DERS [68]	Strategies	4	5	189	.89	9.59 (4.43)	133	.89	9.26 (4.13)
Emotion regulation	DERS [68]	Emotional clarity: lack of emotional clarity	4	2	189	.89	3.66 (1.62)	133	.85	3.53 (1.48)
Psychological distress	K6^j^ [69]	N/A	4	6	189	.82	5.63 (4.11)	133	.81	5.71 (3.98)
Affect	I-PANAS-SF^k^ [70]	Negative affect	5	5	189	.79	9.25 (3.46)	133	.83	9.17 (3.69)
Affect	I-PANAS-SF [70]	Positive affect	5	5	189	.78	15.09 (3.71)	133	.80	15.12 (4.00)
Stress	DASS^l^ [71]	Stress	5	7	189	.86	16.00 (8.76)	133	.81	15.35 (7.73)
Emotion parenting^m^	CTNES-SF^n^ [72]	Emotion dismissing	7	12	189	.69	2.54 (0.80)	133	.72	2.57 (0.81)
Emotion parenting	CTNES-SF [72]	Emotion coaching	7	9	189	.87	6.19 (0.82)	133	.91	6.22 (0.91)
Reflective functioning	PRFQ^o^ [73]	Prementalizing	5	6	189	.53	1.75 (0.66)	133	.72	1.81 (0.79)
Reflective functioning	PRFQ [73]	Certainty about mental states	5	6	189	.78	3.56 (1.11)	133	.77	3.59 (1.09)
Reflective functioning	PRFQ [73]	Interest and curiosity	5	5	189	.76	5.28 (0.91)	133	.87	5.21 (0.96)
Emotion beliefs^p^	PBACE^q^ [74]	Anger	6	6	189	.78	25.06 (4.87)	133	.83	24.97 (5.44)
Emotion beliefs	PBACE [74]	Control	6	5	189	.77	11.99 (4.41)	133	.74	11.89 (4.16)
Emotion beliefs	PBACE [74]	Manipulation	6	4	189	.89	9.96 (4.95)	133	.90	9.65 (5.00)
Emotion beliefs	PBACE [74]	Autonomy	6	7	189	.88	17.52 (6.63)	133	.88	17.78 (6.53)
Emotion beliefs	PBACE [74]	Stability	6	4	189	.64	11.95 (3.65)	133	.71	11.53 (3.78)
Family climate^r^	SEFQ^s^ [75]	Positive emotion expression	9	11	189	.95	7.19 (1.59)	133	.94	7.27 (1.58)
Family climate	SEFQ [75]	Negative emotion expression	9	12	189	.91	3.66 (1.44)	133	.90	3.56 (1.41)
Parental conflict^t^	CCS^u^ [76,77]	Verbal conflict	5	4	189	.86	2.49 (0.70)	122	.83	2.41 (0.68)
Parental conflict	CCS [76,77]	Physical conflict	5	1	189	N/A	0.23 (0.42)	122	N/A	0.16 (0.37)

a MAP-DB: Multidimensional Assessment of Preschool Disruptive Behavior [63].

b SMFQ: Short Mood and Feelings Questionnaire [64].

c N/A: not applicable.

d SCAS: Spence Children Anxiety Scale [65].

e GAD and sep anxiety: Generalized anxiety disorder (3 items) and separation anxiety disorder (1 item) combined.

f EERBQ: Early Emotion Regulation Behavior Questionnaire [66].

g PANAS-C-P: Positive and Negative Affect Schedule for Children [67].

h DERS: Difficulties in Emotion Regulation Scale [68].

i Nonacceptance: Nonacceptance of emotional responses.

j K6: Kessler Psychological Distress Scale [69].

k I-PANAS-SF: short-form International Positive and Negative Affect Scale [70].

l DASS: Depression, Anxiety and Stress Scale [71].

m Emotion parenting: emotion-related parenting.

n CTNES-SF: Coping with Toddlers Negative Emotions Scale—Short Form [72].

o PRFQ: Parental Reflective Functioning Questionnaire [73].

p Emotion beliefs: beliefs about children’s emotions.

q PBACE: Parents’ Beliefs About Children’s Emotions Questionnaire [74].

r Family climate: family emotional climate.

s SEFQ: Self-Expressiveness in the Family Questionnaire [75].

t Parental conflict: interparental conflict.

u CCS: Co-Parental Communications Scale [76,77].

#### EMI Survey Measures

##### Measures Overview

Momentary parent and child emotion dysregulation and negative affect were reported via twice-daily 1-minute pre- and post-EMI surveys at 7:30 AM and 7:30 PM. These prompts did not expire, and parents could respond at their convenience. The twice-daily EMI surveys were identical to each other except for 1 pre-EMI item asking about difficult parenting situations in the previous 24 hours. Item selection and the schedule for delivering survey prompts were informed by a previous EMA study identifying the most suitable state-based items for capturing in-the-moment parent and child emotion dysregulation and negative affect and times at which parents were most likely to respond to prompts [55]. If a participant scored at or above the predetermined cutoff value on any item of the pre-EMI survey, they were eligible to receive a resource. Eligibility thresholds were defined across 4 domains: parent negative affect (≥1), child negative affect (≥1 for anger and ≥2 for sadness), parent emotion dysregulation (≥1), and child emotion dysregulation (≥2). These thresholds were selected to indicate at least mild elevations in negative affect or emotion dysregulation, warranting in-the-moment support.

##### Parent and Child Affect

Parent and child negative affect were measured using 3 items drawn from the short-form International Positive and Negative Affect Schedule (I-PANAS-SF) [70] to capture momentary emotional states (“upset,” “angry,” and “sad”). Items included “Indicate to what extent your child feels this way right now” and “Indicate to what extent you feel this way right now.” Parents rated items on a 10-point scale from 1 (not at all) to 10 (extremely), reflecting how they or their child felt “right now, in this moment.” These state-based items were selected to align with the trait-based I-PANAS-SF included in the baseline and 2-week post surveys, enabling comparison between momentary and general affect. The resource eligibility thresholds for parent and child negative affect were ≥1 for parent negative affect, ≥1 for child anger, and ≥2 for child sadness.

##### Parent and Child Emotion Regulation

Parent and child emotion dysregulation were assessed using 3 items from the state-based version of the Difficulties in Emotion Regulation Scale (S-DERS) [78]. Items included “My emotions feel overwhelming” and “I am having difficulty controlling my behaviours.” As the original S-DERS was developed for adults, 1 item was adapted to assess child emotion regulation (“My child is having difficulty controlling their behaviours.”). Parents rated items on a 10-point scale from 1 (not at all) to 10 (extremely), reflecting their or their child’s current emotional state. The resource eligibility thresholds for parent and child emotion regulation were ≥1 for parent emotion dysregulation and ≥2 for child emotion dysregulation.

### Statistical Analysis

To evaluate feasibility in terms of participant recruitment, retention, and engagement (aim 1), data from the baseline, 2-week post, and EMI surveys were analyzed using descriptive statistics. Pearson pairwise correlations examined associations between demographic characteristics and engagement with pre- and post-EMI surveys and the 2-week post survey. To evaluate acceptability of the program content and delivery method (aim 2), acceptability items from the 2-week post survey were analyzed descriptively to assess participant feedback about the Daily Growth program. To assess feasibility of the research methods in evaluating the efficacy of Daily Growth (aim 3), 2 sets of analyses were conducted in line with the two survey streams: (1) baseline to 2-week post survey outcomes: Bonferroni-adjusted linear regression analyses were conducted for parent and child outcomes. Each model used a postintervention outcome (eg, parent or child emotion regulation and mental health) as the dependent variable and study condition (intervention or control) as the independent variable. Two models were estimated for each outcome: an unadjusted model examining the association between assigned condition and the 2-week post survey outcome, and an adjusted model controlling for the baseline value of the outcome. Effect sizes were also calculated, with Cohen *d* reported for the unadjusted model and η² reported for the adjusted model. (2) EMI outcomes: Post-EMI survey data were analyzed using linear mixed-effects regression models, with condition as the fixed effect and participant as the random effect. Two models were estimated for each outcome: an unadjusted model examining the association between assigned condition and post-EMI outcome, and an adjusted model controlling for the pre-EMI value of the outcome. All analyses were intention-to-treat and used the maximum available data, resulting in variation in sample size per test.

### Protocol Deviations and Technical Issues

Several technical issues occurred during the pilot. First, approximately half of the participants did not receive post-EMI surveys due to a programming error that sent surveys only if participants completed their pre-EMI survey within 15 minutes of the invitation email. This was corrected midway through the trial by removing the time restriction and sending post-EMI surveys to all participants regardless of resource eligibility. For analysis, participants were categorized into group 1 (affected by the issue) and group 2 (unaffected by the issue), with both groups balanced across control and intervention conditions and showing no other differences. Second, a programming oversight allowed participants to complete EMI surveys multiple times; only first completions were retained for analysis. Third, EMI survey prompts did not account for participants’ time zones, resulting in prompts sent outside intended times for participants residing outside Victoria, New South Wales, Tasmania, or the Australian Capital Territory. Finally, 57 EMI survey responses and 2522 Platform O prompts missing participant IDs were excluded from analysis.

## Results

### Feasibility Outcomes

#### Participant Flow

**Figure 2.**
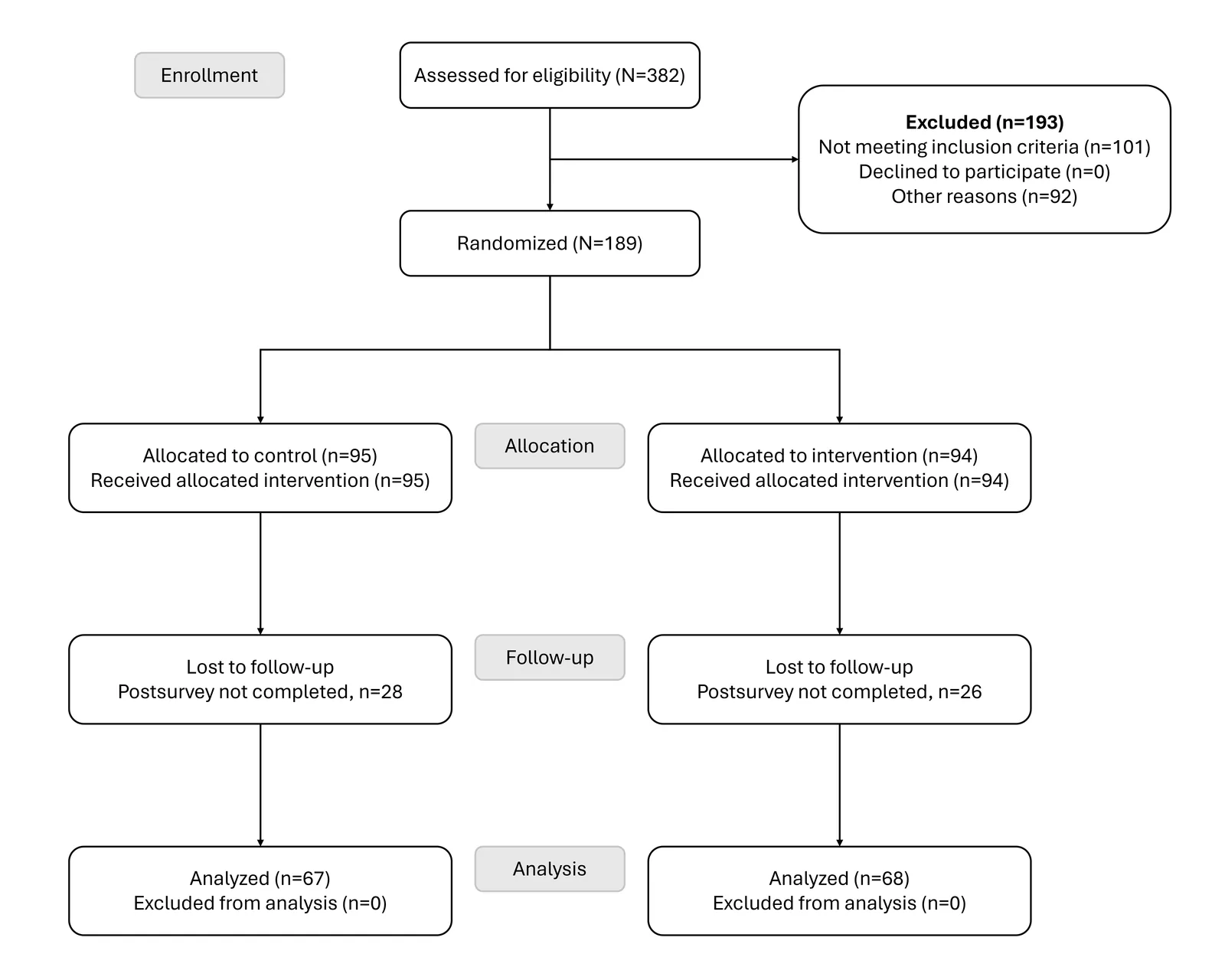
Participant flow through enrollment, screening, baseline completion, 2-week post survey completion, and analysis.

Participant flow is presented in Figure 2. Of the 382 participants assessed for eligibility, approximately half were excluded for reasons including not meeting inclusion criteria, incomplete demographic data, residing outside Australia, being identified as bots, having no EMI prompts assigned, lacking a participant ID, or being duplicate entries. Overall, 71% (135/189) of the participants who completed the baseline survey also completed the 2-week post survey, meeting the prespecified target of <30% attrition.

#### Participant Demographics

Participant demographics are presented in Table 2. The study enrolled 189 parents and carers of children aged 2‐4 years, exceeding the recruitment target of 150 participants. Randomization resulted in groups that were broadly comparable on baseline demographic characteristics. The mean participant age was 36.4 (SD 5.2) years in the control group and 36.7 (SD 4.8) years in the intervention group; the overall sample mean age was 36.6 (SD 5.0) years. Most participants were mothers, and the predetermined recruitment target of 25% fathers was not met. Compared with the most recent census of Australian families [23,79,80], single parents and parents of Aboriginal and Torres Strait Islander descent were overrepresented in the study sample. Parents with a low income, born overseas, who speak another language, and those without a university degree were underrepresented.

**Table 2.** Comparison of demographic characteristics across control and intervention conditions, the total sample, and the Australian population (N=189)^a^.

Variable	Control, n (%)	Intervention, n (%)	Total sample, n (%)	Australian population, %
Participant’s sex				
Female	82 (86.3)	82 (87.2)	164 (86.8)	50.7
Male	13 (13.7)	11 (11.7)	24 (12.7)	49.3
Nonbinary	0 (0)	1 (1.1)	1 (0.5)	—^b^
Child’s sex				
Female	46 (48.4)	47 (50.0)	93 (49.2)	—
Child’s age, years				
2	17 (17.9)	20 (21.3)	37 (19.6)	—
3	29 (30.5)	31 (33.0)	60 (31.7)	—
4	49 (51.6)	43 (45.7)	92 (48.7)	—
Single parent	9 (9.5)	9 (9.6)	18 (9.5)	4.2
Number of children in the home				
1	15 (15.8)	17 (18.1)	32 (16.9)	42
2	56 (58.9)	53 (56.4)	109 (57.7)	39
3	17 (17.9)	20 (21.3)	37 (19.6)	14
4+	7 (7.4)	4 (4.3)	11 (5.8)	5
Level of schooling				
Did not complete high school	8 (8.4)	14 (14.9)	22 (11.6)	20.7
Completed high school	87 (91.6)	80 (85.1)	167 (88.4)	79.3
Highest qualification				
No tertiary qualification	9 (9.5)	4 (4.3)	13 (6.9)	38.8
Trade certificate or diploma	19 (20.0)	20 (21.3)	39 (20.6)	17
Bachelor’s degree	31 (32.6)	38 (40.4)	69 (36.5)	20.3
Postgraduate degree	36 (37.9)	32 (34.0)	68 (36.0)	8.1
Participant born overseas	14 (14.7)	15 (16.0)	29 (15.3)	27.6
Language other than English	8 (8.4)	4 (4.3)	12 (6.3)	22
Aboriginal/Torres Strait Islander	6 (6.3)	4 (4.3)	10 (5.3)	3.8
Low income (AU$ <52,000 per year)	13 (13.7)	13 (13.8)	26 (13.8)	21

a Australian population data are from the Australian Bureau of Statistics [23,79]. Population includes Australian parents living with a dependent child.

b Not available.

#### EMI Completion

**Figure 3.**
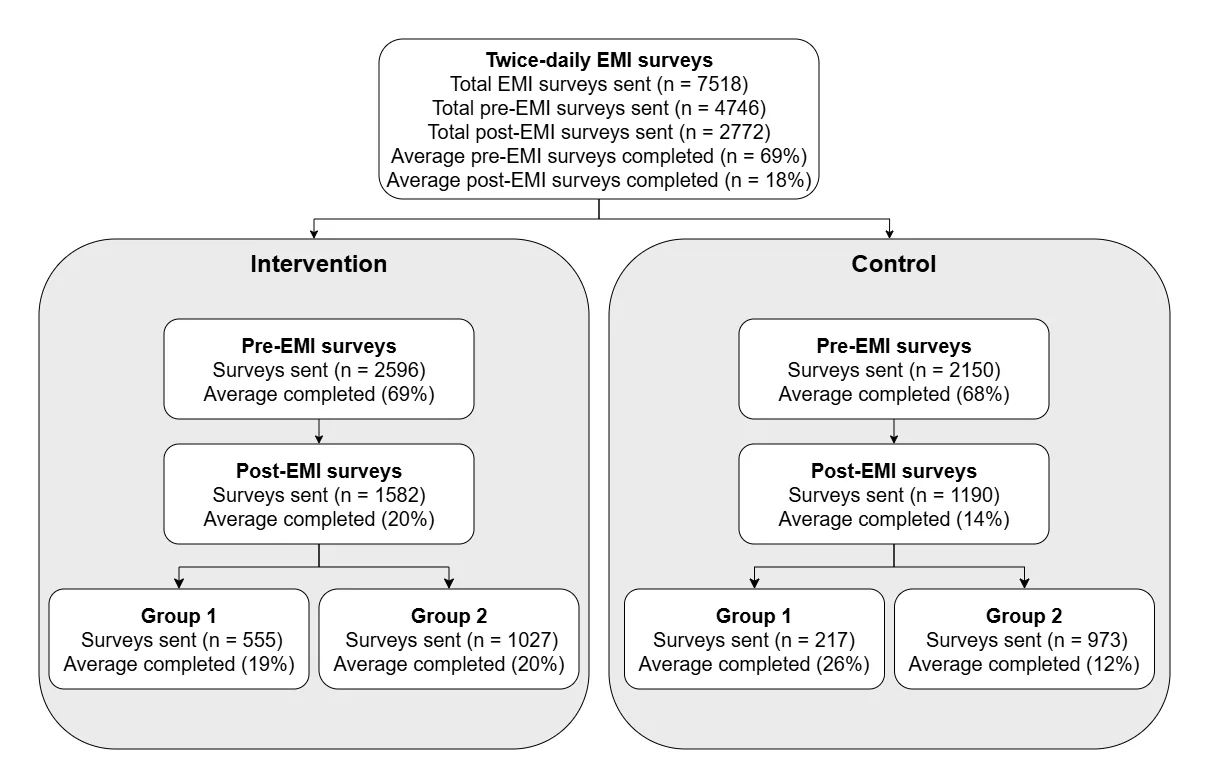
EMI survey completion rates across intervention and control conditions. Technical issues affected group 1 (ie, the post-EMI survey was not consistently sent). No technical issues affected group 2. EMI: ecological momentary intervention.

**Figure 4.**
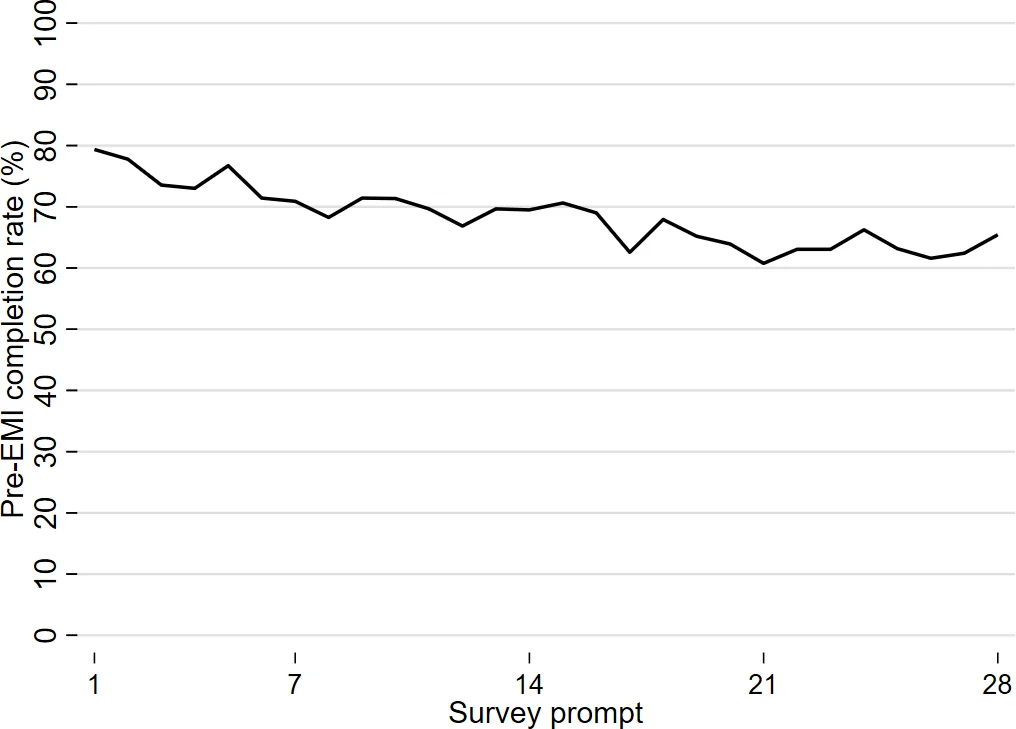
Average pre-EMI survey completion rates by survey time point across the 14-day intervention period. EMI: ecological momentary intervention.

EMI completion rates over the 2-week study period are presented in Figure 3. On average, participants completed close to two-thirds of the pre-EMI surveys, exceeding the prespecified engagement target of 50%. However, post-EMI survey completion was lower and fell below the prespecified 50% target. Completion rates did not differ substantially between conditions. For pre-EMI surveys, the majority of the participants (57.7%) completed at least 75% of surveys. Around 1 in 6 (17.5%) participants completed fewer than 25% of pre-EMI surveys. In contrast, post-EMI survey completion was low, with most participants (58.2%) completing fewer than 25% of surveys, and only about 1 in 10 (9%) completing at least 75%. Twenty-three participants in group 1 (ie, affected by technical issues) did not receive any post-EMI survey prompts. Figure 4 presents the average pre-EMI survey completion rates across study time points. Completion rates fluctuated between 60% and 80% across the 2-week period.

#### Demographic Characteristics and Engagement

Prespecified Pearson pairwise correlations were conducted to examine associations between pre-EMI survey completion rates and participant demographic characteristics. There was evidence for a small association between participants’ pre-EMI survey completion rate and child’s age (*r*=0.15; *P*=.046), suggesting that completion rates were slightly higher among parents of older children. However, we found no evidence to suggest associations between any other demographic characteristics and pre-EMI completion rates.

#### Resource Eligibility

**Figure 5.**
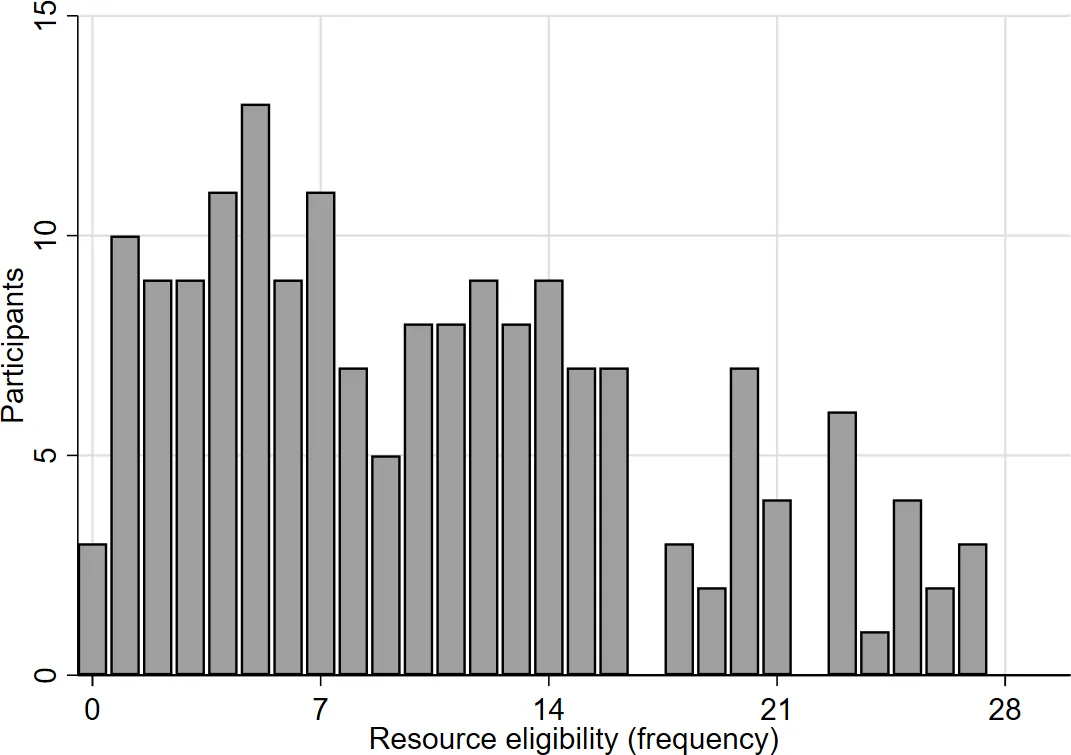
Frequency distribution of resource eligibility per participant by day of participation over 2 weeks.

Figure 5 presents the number of survey responses per participant that were eligible to receive a resource (ie, scored above the cutoff on the pre-EMI survey). Among all completed pre-EMI surveys, 56% met criteria for receiving a resource, with an average of 43% eligible pre-EMI surveys per participant. On average, parenting support was offered once per day based on the pre-EMI survey cutoff.

### Acceptability

Program acceptability results are shown in Table 3. Interpretation of acceptability outcomes was limited by an error in the survey response options, as both ends of the scale were labeled “Strongly disagree,” (ie, participants were shown as Strongly disagree, Disagree, Neutral, Agree, and Strongly disagree), meaning that it was not possible to determine whether participants intended to select the lowest or highest level of acceptability. It is also likely that responses are skewed, as participants who would have selected “Strongly agree” may instead have selected “Agree,” given that the “Strongly agree” option was not available. Notably, only a small number of participants selected either “Strongly disagree” response option. For example, for the item “Overall, I am satisfied with the Daily Growth program.” only 3% (2/67) selected “Strongly disagree.”

As shown in Table 3, the majority of intervention participants who completed the acceptability survey found the Emotion Coaching and Active Play resources useful, with over two-thirds agreeing that the program was easy to use. Just less than half of the participants felt that Daily Growth met their needs, and more than half reported overall satisfaction. Almost half of the participants indicated that the program helped them manage challenging parenting situations, while more than half reported that it improved their understanding of their child’s emotions. For nearly half of the participants, the program also strengthened their bond with their child. Additionally, two-thirds planned to continue using the skills they learned. Most participants (94%) reported that the Daily Growth resources were about the right length, and the majority (79%) indicated that they would recommend the program to other parents. However, consistent with the technical issues described earlier, approximately one-third of the participants (31%) reported encountering issues while using the app, most commonly relating to not receiving some EMI surveys.

**Table 3.** Acceptability of Daily Growth at 2-week post survey (N=67).

Item	Strongly disagree^a^, n (%)		Disagree, n (%)		Neutral, n (%)		Agree, n (%)		Strongly disagree^a^, n (%)	
I am satisfied with Daily Growth	0 (0)		10 (14.9)		15 (22.4)		40 (59.7)		2 (3)	
Daily Growth meets my needs	1 (1.5)		13 (19.4)		21 (31.3)		30 (44.8)		2 (3)	
I found Emotion Coaching useful	0 (0)		1 (1.5)		20 (29.9)		43 (64.2)		3 (4.5)	
I found Active Play useful	0 (0)		1 (1.5)		27 (40.3)		36 (53.7)		3 (4.5)	
Daily Growth was easy to use	0 (0)		7 (10.5)		10 (14.9)		45 (67.2)		5 (7.5)	
I will use the skills I learned	0 (0)		3 (4.5)		14 (20.9)		46 (68.7)		4 (6)	
Daily Growth helped me...										
parent with partner	4 (6)		17 (25.4)		23 (34.3)		21 (31.3)		2 (3)	
manage parenting challenges	1 (1.5)		7 (10.5)		27 (40.3)		29 (43.3)		3 (4.5)	
understand child’s emotions	0 (0)		9 (13.4)		15 (22.4)		39 (58.2)		4 (6)	
strengthen parent-child bond	0 (0)		9 (13.4)		26 (38.8)		30 (44.8)		2 (3)	

a Due to a survey error, both ends of the acceptability scale were labeled “Strongly disagree,” limiting interpretation of responses. It is possible that some participants selected “Agree” in place of “Strongly agree,” since the latter option was not available. Few participants overall selected “Strongly disagree.”

### Descriptive Efficacy

#### Two-Week Post survey Outcomes

Descriptive statistics for the outcome measures are reported in Table 4. Internal consistency (Cronbach α) for most measures was similar to values reported in the original validation studies [63-66,68-75,81]. Multivariable regression analyses examining condition differences are also presented in Table 4. Two models were estimated for each outcome: an unadjusted model and a model adjusted for baseline values. Findings were largely consistent across models, with significant results differing only for overall parent emotion regulation. Specifically, the between-group difference in overall parent emotion regulation was statistically significant in the unadjusted model but did not remain significant after adjusting for baseline scores. Overall, results indicated greater improvements from baseline to 2-week postintervention in the intervention group than in the control group in parent emotion dysregulation, including nonacceptance of emotional responses, parent psychological distress, and parent negative affect. No significant improvements were evident for any child outcomes.

**Table 4.** Associations between condition status and parent and child 2-week post survey outcomes^a^.

Outcome	Unadjusted					Adjusted for baseline				
	Effect size^b^	*B* value	LL^c^	UL^d^	*P* value	Effect size^e^	*B*	LL	UL	*P* value
Child outcomes										
Behavioral problems										
Temper loss	0.20	−0.17	−0.47	0.13	.26	0.00	0.03	−0.20	0.25	.81
Noncompliance	0.10	−0.11	−0.45	0.24	.54	0.00	−0.02	−0.28	0.23	.87
Aggression	0.23	−0.21	−0.52	0.10	.19	0.00	0.02	−0.20	0.24	.86
Depression	0.30	−0.92	−1.95	0.12	.08	0.02	−0.72	−1.54	0.10	.09
Anxiety	−0.05	0.10	−0.56	0.76	.77	0.00	0.17	−0.39	0.73	.55
Emotion regulation										
Mindfulness	0.08	−0.11	−0.61	0.38	.66	0.00	−0.11	−0.50	0.28	.59
Verbal help-seeking	−0.17	0.27	−0.26	0.81	.32	0.01	0.19	−0.18	0.57	.31
Verbal venting	−0.11	0.14	−0.31	0.60	.53	0.01	0.22	−0.12	0.56	.21
Physical venting	−0.13	0.19	−0.31	0.69	.45	0.02	0.29	−0.06	0.64	.10
Emotional reactivity	0.26	−0.25	−0.58	0.08	.14	0.00	−0.09	−0.31	0.14	.45
Negative affect	−0.05	0.15	−0.85	1.15	.77	0.01	0.38	−0.47	1.23	.37
Parent outcomes										
Emotion regulation										
Total	0.37	−4.84	−9.36	−0.32	.04	0.01	−1.87	−4.62	0.89	.18
Nonacceptance^f^	0.49	−1.49	−2.53	−0.45	.01	0.04	−0.90	−1.65	−0.15	.02
Goals	0.22	−0.62	−1.61	0.36	.21	0.00	−0.25	−0.97	0.46	.49
Impulse control difficulties	0.26	−1.16	−2.71	0.38	.14	0.01	−0.50	−1.53	0.54	.35
Strategies	0.29	−1.19	−2.60	0.21	.10	0.00	−0.16	−1.04	0.72	.72
Emotional clarity^g^	0.25	−0.37	−0.87	0.14	.15	0.02	−0.31	−0.71	0.08	.12
Psychological distress	0.40	−1.57	−2.92	−0.23	.02	0.04	−0.91	−1.71	−0.11	.03
Positive affect	0.01	−0.03	−1.41	1.35	.96	0.01	0.66	−0.34	1.65	.19
Negative affect	0.39	−1.40	−2.65	−0.15	.03	0.04	−0.94	−1.72	−0.16	.02
Stress	0.20	−1.52	−4.17	1.13	.26	0.02	−1.58	−3.50	0.33	.11
Emotion-related parenting										
Emotion coaching	−0.03	0.03	−0.28	0.35	.84	0.00	0.01	−0.24	0.25	.97
Emotion dismissing	0.18	−0.15	−0.43	0.13	.29	0.00	−0.09	−0.31	0.14	.44
Reflective functioning										
Prementalizing	−0.02	0.02	−0.25	0.29	.90	0.00	0.04	−0.16	0.24	.68
Certainty	−0.13	0.15	−0.23	0.52	.44	0.00	0.10	−0.18	0.38	.48
Interest and curiosity	0.00	0.00	−0.33	0.33	.99	0.02	0.21	−0.05	0.47	.12
Emotion beliefs^h^										
Anger	−0.10	0.57	−1.30	2.44	.55	0.00	0.16	−1.12	1.45	.80
Control	0.17	−0.69	−2.12	0.74	.34	0.00	−0.38	−1.43	0.68	.48
Manipulation	0.08	−0.39	−2.10	1.33	.66	0.00	0.31	−0.80	1.42	.58
Autonomy	0.08	−0.52	−2.77	1.73	.65	0.00	−0.44	−2.02	1.13	.58
Stability	0.28	−1.06	−2.35	0.23	.11	0.00	−0.34	−1.47	0.79	.55
Family climate^i^										
Positive emotion expression	−0.10	0.15	−0.39	0.69	.58	0.00	0.01	−0.32	0.35	.94
Negative emotion expression	0.05	−0.07	−0.55	0.42	.79	0.00	−0.06	−0.39	0.28	.74
Verbal conflict	0.16	−0.11	−0.35	0.13	.37	0.01	−0.08	−0.24	0.08	.31
Physical conflict	0.00	0.00	−0.05	0.05	≥.99	0.00	0.01	−0.04	0.05	.81

a Participant condition status, that is, their assignment to either the intervention or control condition, was entered as the independent variable. Two models were estimated: an unadjusted model and a model adjusted for the corresponding baseline value.

b Effect size for adjusted models=η².

c LL: lower limit.

d UL: upper limit.

e Effect size for unadjusted models=Cohen *d*.

f Nonacceptance: nonacceptance of emotional responses.

g Emotional clarity: lack of emotional clarity.

h Emotion beliefs: beliefs about children’s emotions.

i Family climate: family emotional climate.

#### In-the-Moment Post-EMI Outcomes

Table 5 presents the linear mixed model regression analysis of post-EMI survey outcomes. Two models were estimated for each outcome: an unadjusted model and a model adjusted for corresponding pre-EMI values. Findings were consistent across both models, demonstrating a significant association between condition and momentary parent negative affect. No additional significant effects were found. Intraclass correlation values indicated that a small proportion of variance in outcome measures was attributable to individual differences.

**Table 5.** Associations between condition status and parent and child post–ecological momentary intervention survey outcomes^a^.

Outcome	Unadjusted					Adjusted for pre-EMI^b^				
	*B* value	LL^c^	UL^d^	*P* value	ICC^e^	*B* value	LL	UL	*P* value	ICC
Child outcomes										
Emotion regulation^f^	0.31	−0.30	0.93	.314	0.33	−0.11	−0.53	0.32	.615	0.10
Negative affect										
Sad	0.00	−0.48	0.49	.990	0.14	−0.03	−0.44	0.38	.872	0.05
Angry	0.11	−0.33	0.54	.634	0.25	−0.05	−0.37	0.26	.732	0.13
Parent outcomes										
Emotion regulation										
Overwhelming^g^	−0.33	−0.80	0.15	.176	0.37	−0.23	−0.49	0.04	.099	0.11
Behaviors^h^	−0.23	−0.71	0.25	.347	0.58	−0.17	−0.51	0.16	.305	0.41
Negative affect (upset)	−0.50	−0.95	−0.05	*.029*	0.48	−0.37	−0.71	−0.04	.*028*	0.32

a Participant condition status, that is, their assignment to either the intervention or control condition, was entered as the independent variable. Two models were estimated: an unadjusted model and a model adjusted for the corresponding pre-EMI value.

b EMI: ecological momentary intervention.

c LL: 95% CI lower limit.

d UL: 95% CI upper limit.

e ICC: intraclass correlation.

f “My child is having difficulty controlling their behaviours” EMI item.

g “My emotions are overwhelming” EMI item.

h “I am having difficulty controlling my behaviors” EMI item.

## Discussion

### Principal Findings

This pilot randomized controlled trial evaluated the feasibility, engagement, and acceptability of a small-scale prototype of the Daily Growth parenting program, a digital intervention designed to provide in-the-moment support for parents using an EMI approach. Overall, the study met its recruitment target and demonstrated a high retention rate. Engagement with pre-EMI surveys exceeded expectations, translating to parents on average completing a survey once per day. Participants responded positively to the program and found the resources useful. However, post-EMI survey completion rates were lower than anticipated, with parents responding approximately 1 in 5 times, and certain demographic groups were underrepresented. Together, these findings offer valuable insights for refining the program and guiding future research.

### Feasibility

This pilot study exceeded the recruitment target of 150 participants and retention rates were high compared with similar parenting interventions [20,82,83], indicating a high level of participant interest and engagement. This may be attributed to Daily Growth’s user-centered design, including flexible and tailored support based on parent needs. Nevertheless, some attrition occurred between screening and baseline, as well as during the 2-week intervention period, highlighting opportunities to refine the enrollment process and strengthen engagement strategies. Engagement with pre-EMI surveys exceeded expectations. Overall, engagement with the daily prompts throughout the study period was at a similar level to other EMA or intervention studies [23] and close to the recommended rate of 80% [10]. Notably, the predefined engagement benchmark for this study was 50%, as Daily Growth is intended to be a light-touch, accessible program that would not be overly burdensome for busy parents. Achieving engagement levels substantially above this target therefore provides strong preliminary support for the acceptability of the delivery schedule. However, engagement with post-EMI surveys was lower than the target of 50% completion, which may be attributed to having separate online systems. As EMI survey prompts were delivered via email, while surveys and videos were delivered through Platform O, it is possible that once parents completed the pre-EMI survey and were directed to a video or website elsewhere, they did not return to their email to click on the post-EMI survey prompt. Using a smartphone app with push notifications could help streamline this process by keeping all prompts and resources within a single, integrated system. Additionally, providing onboarding instructions to emphasize the importance of completing all survey components may further support engagement. It is also important to note that technical issues affected the post-EMI surveys and should be addressed in future implementations.

This pilot study demonstrated Daily Growth’s potential to engage a diverse group of parents, an ongoing challenge in online parenting intervention studies [20,84]. However, fathers, migrant parents, and parents with a low level of education were underrepresented, consistent with patterns in previous intervention research [20,82-86]. Fathers may perceive parenting programs as less relevant to them or feel that their contributions are undervalued, while migrant parents may face barriers such as language, accessibility, or differing cultural expectations [20,82-86]. Although the primary focus of this pilot was not on recruiting a representative sample but rather on testing the technical feasibility and engagement with the Daily Growth prototype, future iterations should aim to increase inclusivity by incorporating father-specific recruitment strategies and ensuring that program materials are broadly accessible to better reach underrepresented groups.

### Acceptability

Participants responded positively to Daily Growth, with most finding the resources useful and willing to recommend the program to others. These findings suggest that the Design Mapping framework and EMI approach successfully enhanced parent satisfaction, consistent with previous studies finding these approaches acceptable [13,37,87-91]. Approximately half of the participants reported that the program fully met their needs or helped them manage parenting challenges, a reasonable outcome given the pilot offered only 10 videos covering 5 parenting situations. The full Daily Growth program, featuring 90 video resources across 3 program types, will address a broader range of parenting challenges and better accommodate diverse family needs. Participants also reported technical difficulties, particularly with post-EMI survey prompts. Improving technical infrastructure to streamline user experience and minimize disruptions remains a priority for future iterations.

### Descriptive Outcomes

Descriptive outcome data were collected to test the feasibility of the program evaluation methods, more specifically to assess whether the measures are sensitive to change, whether outcome data can be collected completely, and whether the measurements are viable to use in a larger trial. The prototype version of Daily Growth showed significant reductions in momentary negative affect (EMI outcomes) in participants in the intervention condition compared with the control, suggesting that the program’s real-time delivery may effectively support parents during challenging situations. These findings align with previous emotion-focused parenting interventions [92-95] and EMI-based parenting programs [13,37] and indicate that brief, targeted support can help parents manage their emotions more effectively. Furthermore, baseline to 2-week postintervention improvements in parent emotion regulation and mental health outcomes were observed, including reduced nonacceptance of emotional responses, psychological distress, and negative affect.

Notably, the between-group difference in total parent emotion dysregulation was significant in the unadjusted model but did not remain significant after adjusting for baseline scores. This likely reflects the strong stability of emotion regulation over time and limited statistical power in this pilot sample. Furthermore, no significant effects emerged for baseline to 2-week post survey child outcomes or broader parenting behaviors such as emotion coaching. The program was designed to target parent emotion regulation and parenting behaviors; hence, child outcomes represent more distal targets. Given the pilot’s brief duration (2 weeks), limited intervention content (only 10 of the total 60 videos were available), and small sample size, detecting changes in these outcomes was not the focus of the current trial. Child emotion regulation develops gradually through repeated parent-child interactions, and meaningful shifts in parenting practices typically require sustained exposure to intervention content. The full 6-week Daily Growth program, with substantially more content and longer intervention exposure, provides a more appropriate test of child-level impacts in a larger, adequately powered trial.

### Implications for Future Research

Several directions for future research and program refinement should be considered. First, investigating how parent gender influences program efficacy warrants attention, as mothers and fathers often differ in their parenting approaches [96-98], suggesting opportunities for targeted tailoring. Second, although baseline parental mental health was assessed, this pilot study was not powered to examine whether feasibility, acceptability, or descriptive efficacy outcomes differed by mental health status. Future trials could explore mental health as a potential moderator of engagement and program effects, as these parents may have greater needs for support and may engage differently with EMI program delivery [99]. Furthermore, optimizing intervention timing deserves careful consideration. While in-the-moment delivery can be highly effective [29,100], some parents may be more receptive to learning new skills when not experiencing high emotional dysregulation [101]. Future research should systematically examine timing and delivery strategies that maximize both learning and immediate support, potentially offering parents flexibility when they access content.

### Strengths and Limitations

This study makes several important contributions. The randomized controlled trial design minimizes bias and provides a robust test of full trial methods. Most notably, this represents one of the first applications of an EMI approach to digital parenting interventions. Beyond enhancing engagement through regular prompts, the EMI design generated valuable real-time data on parents’ momentary experiences and immediate responses to brief video interventions. These findings provide early support for EMI’s viability in early childhood parenting support and demonstrate that twice-daily prompts are generally acceptable to parents. However, several limitations warrant consideration. First, the pilot tested only a 2-week prototype, whereas the full program spans 6 weeks. While short-term feasibility was demonstrated, sustained engagement with twice-daily prompts over extended periods remains uncertain. Second, the sample, although adequate for pilot testing, was relatively small and underrepresented fathers, migrant parents, and those with lower educational attainment, limiting generalizability. A third limitation is that the prototype platform was not able to capture the number of resources watched by each participant, meaning that intervention exposure could not be quantified beyond the EMI survey data. Technical issues also affected data quality and user experience. Approximately half of the participants did not receive post-EMI survey prompts due to a programming error, and prompts did not account for time zones, resulting in some participants receiving surveys outside intended times. In addition, multiple completions of EMI surveys and missing participant IDs resulted in the exclusion of a portion of responses. These issues highlight the importance of refining the technical infrastructure prior to a fully powered trial. Finally, the limited intervention content (10 videos covering 5 situations) constrained our ability to detect changes in child outcomes or broader parenting behaviors, which likely require more comprehensive exposure to demonstrate effects.

### Conclusions

Reaching diverse families and sustaining their engagement remains one of the greatest challenges for universal parenting programs. This pilot study demonstrates that delivering parenting support through an EMI approach is both feasible and acceptable. Testing a small-scale Daily Growth prototype, we found that brief, tailored video resources successfully engaged parents in real time. High recruitment and retention rates, combined with positive parent feedback and engagement exceeding expectations for pre-EMI surveys, provide compelling evidence that tailored EMI approaches can overcome traditional barriers to reach and engagement in digital parenting programs. However, low post-EMI completion rates indicate the need for refinements to reduce participant burden and improve response rates. These promising findings establish a foundation for a larger trial to rigorously assess efficacy, optimize delivery methods, and enhance accessibility for underrepresented groups, particularly fathers and migrant parents. Beyond demonstrating feasibility, this study advances digital parenting interventions through its innovative co-design approach. Daily Growth represents a new model for scalable, data-driven parenting support that has the potential to improve parenting practices and child mental health outcomes at a population level.

## Supplementary material

10.2196/87917 Checklist 1 CONSORT-EHEALTH checklist.

## Abbreviations

EMA: ecological momentary assessment
EMI: ecological momentary intervention
I-PANAS-SF: short-form International Positive and Negative Affect Schedule
S-DERS: state-based version of the Difficulties in Emotion Regulation Scale
