# Supplementary material for: “Daily Growth” Parenting Program Delivered via Ecological Momentary Intervention: Pilot Randomized Controlled Trial of a Prototype
Source: JMIR Pediatr Parent. 2026 Sep 14;9:e87917. doi: 10.2196/87917 (PMC13574310; doi:10.2196/87917)
Supplement: Supplementary file 1 [file pediatrics-v9-e87917-s001.pdf]

CONSORT-EHEALTH (V 1.6.1) - Submission/Publication Form

The CONSORT-EHEALTH checklist is intended for authors of randomized trials evaluating web-based and Internet-based applications/interventions, including mobile interventions, electronic games (incl multiplayer games), social media, certain telehealth applications, and other interactive and/or networked electronic applications. Some of the items (e.g. all subitems under item 5 - description of the intervention) may also be applicable for other study designs.

The goal of the CONSORT EHEALTH checklist and guideline is to be
a) a guide for reporting for authors of RCTs,
b) to form a basis for appraisal of an ehealth trial (in terms of validity)

CONSORT-EHEALTH items/subitems are MANDATORY reporting items for studies published in the Journal of Medical Internet Research and other journals / scientific societies endorsing the checklist.

Items numbered 1., 2., 3., 4a., 4b etc are original CONSORT or CONSORT-NPT (non-pharmacologic treatment) items.
Items with Roman numerals (i., ii, iii, iv etc.) are CONSORT-EHEALTH extensions/clarifications.

As the CONSORT-EHEALTH checklist is still considered in a formative stage, we would ask that you also RATE ON A SCALE OF 1-5 how important/useful you feel each item is FOR THE PURPOSE OF THE CHECKLIST and reporting guideline (optional).

Mandatory reporting items are marked with a red *.
In the textboxes, either copy & paste the relevant sections from your manuscript into this form - please include any quotes from your manuscript in QUOTATION MARKS,
or answer directly by providing additional information not in the manuscript, or elaborating on why the item was not relevant for this study.

YOUR ANSWERS WILL BE PUBLISHED AS A SUPPLEMENTARY FILE TO YOUR PUBLICATION IN JMIR AND ARE CONSIDERED PART OF YOUR PUBLICATION (IF ACCEPTED).
Please fill in these questions diligently. Information will not be copyedited, so please use proper spelling and grammar, use correct capitalization, and avoid abbreviations.

DO NOT FORGET TO SAVE AS PDF _AND_ CLICK THE SUBMIT BUTTON SO YOUR ANSWERS ARE IN OUR DATABASE !!!

Citation Suggestion (if you append the pdf as Appendix we suggest to cite this paper in the caption):
Eysenbach G, CONSORT-EHEALTH Group
CONSORT-EHEALTH: Improving and Standardizing Evaluation Reports of Web-based and Mobile Health Interventions
J Med Internet Res 2011;13(4):e126

URL: http://www.jmir.org/2011/4/e126/
doi: 10.2196/jmir.1923
PMID: 22209829

stefka.ewald@gmail.com Konto wechseln

Nicht freigegeben

Entwurf gespeichert

* Gibt eine erforderliche Frage an

Your name *

First Last

Stefanie Ewald

Primary Affiliation (short), City, Country *

University of Toronto, Toronto, Canada

Deakin University, Melbourne, Australia

Your e-mail address *

abc@gmail.com

s222210663@deakin.edu.au

Title of your manuscript *

Provide the (draft) title of your manuscript.

Pilot Randomised Controlled Trial of a Prototype Emotion Socialisation Parenting Program: An Ecological Momentary Intervention

Name of your App/Software/Intervention *

If there is a short and a long/alternate name, write the short name first and add the long name in brackets.

Daily Growth

Evaluated Version (if any)

e.g. "V1", "Release 2017-03-01", "Version 2.0.27913"

Prototype

Language(s) *

What language is the intervention/app in? If multiple languages are available, separate by comma (e.g. "English, French")

English

URL of your Intervention Website or App

e.g. a direct link to the mobile app on app in appstore (itunes, Google Play), or URL of the website. If the intervention is a DVD or hardware, you can also link to an Amazon page.

Meine Antwort

URL of an image/screenshot (optional)

Meine Antwort

Accessibility *

Can an enduser access the intervention presently?

- ○ access is free and open
- ○ access only for special usergroups, not open
- ○ access is open to everyone, but requires payment/subscription/in-app purchases
- ◉ app/intervention no longer accessible
- ○ Sonstiges:

Primary Medical Indication/Disease/Condition *

e.g. "Stress", "Diabetes", or define the target group in brackets after the condition, e.g. "Autism (Parents of children with)", "Alzheimers (Informal Caregivers of)"

Universal (Parents of a child aged 2-4)

Primary Outcomes measured in trial *

comma-separated list of primary outcomes reported in the trial

Feasibility (Recruitment, Retention, Engageme

Secondary/other outcomes

Are there any other outcomes the intervention is expected to affect?

Preliminary Efficacy (Parent and Child Emotion Regulation, Parent and Child Mental Health, Emotion Coaching Parenting Practices)

Recommended "Dose" *

What do the instructions for users say on how often the app should be used?

- (x) Approximately Daily
- ( ) Approximately Weekly
- ( ) Approximately Monthly
- ( ) Approximately Yearly
- ( ) "as needed"
- ( ) Sonstiges:

Approx. Percentage of Users (starters) still using the app as recommended after 3 months *

- (x) unknown / not evaluated
- ( ) 0-10%
- ( ) 11-20%
- ( ) 21-30%
- ( ) 31-40%
- ( ) 41-50%
- ( ) 51-60%
- ( ) 61-70%
- ( ) 71%-80%
- ( ) 81-90%
- ( ) 91-100%
- ( ) Sonstiges:

Overall, was the app/intervention effective? *

- ( ) yes: all primary outcomes were significantly better in intervention group vs control
- ( ) partly: SOME primary outcomes were significantly better in intervention group vs control
- ( ) no statistically significant difference between control and intervention
- ( ) potentially harmful: control was significantly better than intervention in one or more outcomes
- ( ) inconclusive: more research is needed
- (•) Sonstiges: The primary aim of this trial was to assess feasibility and accepta

Article Preparation Status/Stage *

At which stage in your article preparation are you currently (at the time you fill in this form)

- ( ) not submitted yet - in early draft status
- (•) not submitted yet - in late draft status, just before submission
- ( ) submitted to a journal but not reviewed yet
- ( ) submitted to a journal and after receiving initial reviewer comments
- ( ) submitted to a journal and accepted, but not published yet
- ( ) published
- ( ) Sonstiges:

Journal *

If you already know where you will submit this paper (or if it is already submitted), please provide the journal name (if it is not JMIR, provide the journal name under "other")

- ○ not submitted yet / unclear where I will submit this
- ○ Journal of Medical Internet Research (JMIR)
- ○ JMIR mHealth and UHealth
- ○ JMIR Serious Games
- ○ JMIR Mental Health
- ○ JMIR Public Health
- ○ JMIR Formative Research
- ◉ Other JMIR sister journal
- ○ Sonstiges:

Is this a full powered effectiveness trial or a pilot/feasibility trial? *

- ◉ Pilot/feasibility
- ○ Fully powered

Manuscript tracking number *

If this is a JMIR submission, please provide the manuscript tracking number under "other" (The ms tracking number can be found in the submission acknowledgement email, or when you login as author in JMIR. If the paper is already published in JMIR, then the ms tracking number is the four-digit number at the end of the DOI, to be found at the bottom of each published article in JMIR)

- ◉ no ms number (yet) / not (yet) submitted to / published in JMIR
- ○ Sonstiges:

TITLE AND ABSTRACT

1a) TITLE: Identification as a randomized trial in the title

1a) Does your paper address CONSORT item 1a? *

I.e does the title contain the phrase "Randomized Controlled Trial"? (if not, explain the reason under "other")

- (●) yes
- ( ) Sonstiges:

1a-i) Identify the mode of delivery in the title

Identify the mode of delivery. Preferably use “web-based” and/or “mobile” and/or “electronic game” in the title. Avoid ambiguous terms like “online”, “virtual”, “interactive”. Use “Internet-based” only if Intervention includes non-web-based Internet components (e.g. email), use “computer-based” or “electronic” only if offline products are used. Use “virtual” only in the context of “virtual reality” (3-D worlds). Use “online” only in the context of “online support groups”. Complement or substitute product names with broader terms for the class of products (such as “mobile” or “smart phone” instead of “iphone”), especially if the application runs on different platforms.

	1	2	3	4	5	
subitem not at all important	○	○	○	○	○	essential

Does your paper address subitem 1a-i? *

Copy and paste relevant sections from manuscript title (include quotes in quotation marks "like this" to indicate direct quotes from your manuscript), or elaborate on this item by providing additional information not in the ms, or briefly explain why the item is not applicable/relevant for your study

Yes, the title states that it is an Ecological Momentary Intervention ("Pilot Randomised Controlled Trial of a Prototype Emotion Socialisation Parenting Program: An Ecological Momentary Intervention").

1a-ii) Non-web-based components or important co-interventions in title

Mention non-web-based components or important co-interventions in title, if any (e.g., “with telephone support”).

	1	2	3	4	5	
subitem not at all important	◯	◯	◯	◯	◯	essential

Does your paper address subitem 1a-ii?

Copy and paste relevant sections from manuscript title (include quotes in quotation marks "like this" to indicate direct quotes from your manuscript), or elaborate on this item by providing additional information not in the ms, or briefly explain why the item is not applicable/relevant for your study

There are no non-web-based components.

1a-iii) Primary condition or target group in the title

Mention primary condition or target group in the title, if any (e.g., “for children with Type I Diabetes”) Example: A Web-based and Mobile Intervention with Telephone Support for Children with Type I Diabetes: Randomized Controlled Trial

	1	2	3	4	5	
subitem not at all important	◯	◯	◯	◯	◯	essential

Does your paper address subitem 1a-iii? *

Copy and paste relevant sections from manuscript title (include quotes in quotation marks "like this" to indicate direct quotes from your manuscript), or elaborate on this item by providing additional information not in the ms, or briefly explain why the item is not applicable/relevant for your study

Yes, it states that this is a parenting program - however, it is a universal program, so it targets all parents and not a specific group ("Pilot Randomised Controlled Trial of a Prototype Emotion Socialisation Parenting Program: An Ecological Momentary Intervention").

1b) ABSTRACT: Structured summary of trial design, methods, results, and conclusions

NPT extension: Description of experimental treatment, comparator, care providers, centers, and blinding status.

1b-i) Key features/functionalities/components of the intervention and comparator in the METHODS section of the ABSTRACT

Mention key features/functionalities/components of the intervention and comparator in the abstract. If possible, also mention theories and principles used for designing the site. Keep in mind the needs of systematic reviewers and indexers by including important synonyms. (Note: Only report in the abstract what the main paper is reporting. If this information is missing from the main body of text, consider adding it)

	1	2	3	4	5	
subitem not at all important	○	○	○	○	○	essential

Does your paper address subitem 1b-i? *

Copy and paste relevant sections from the manuscript abstract (include quotes in quotation marks "like this" to indicate direct quotes from your manuscript), or elaborate on this item by providing additional information not in the ms, or briefly explain why the item is not applicable/relevant for your study

Yes, the abstract includes all the key components of the program (EMI, twice-daily prompts, 3-minute videos, tailoring, Emotion Coaching, Active Play; "Daily Growth is a digital parenting program designed to improve parent and child emotion regulation using an ecological momentary intervention (EMI) approach. Daily Growth sends twice-daily prompts and delivers 3-minute videos tailored to a recent parenting situation, offering three program types to meet different parent needs/preferences... This pilot aimed to assess the feasibility and acceptability of the EMI design and a small prototype of Daily Growth, offering two of the three programs (Emotion Coaching; Active Play) via 10 of 90 planned videos").

1b-ii) Level of human involvement in the METHODS section of the ABSTRACT

Clarify the level of human involvement in the abstract, e.g., use phrases like "fully automated" vs. "therapist/nurse/care provider/physician-assisted" (mention number and expertise of providers involved, if any). (Note: Only report in the abstract what the main paper is reporting. If this information is missing from the main body of text, consider adding it)

	1	2	3	4	5	
subitem not at all important	○	○	○	○	○	essential

Does your paper address subitem 1b-ii?

Copy and paste relevant sections from the manuscript abstract (include quotes in quotation marks "like this" to indicate direct quotes from your manuscript), or elaborate on this item by providing additional information not in the ms, or briefly explain why the item is not applicable/relevant for your study

Yes, we state in the Methods section of the abstract that baseline and post-surveys were completed online, participants received twice-daily EMI surveys via email, and received the resources in a video format ("Participants completed a 25-minute online survey at baseline and two-weeks' post. Over two weeks, participants received twice-daily 1-minute EMI surveys via email. If they reported any parent or child negative affect or emotion dysregulation, they were randomised in-the-moment to receive an online Emotion Coaching or Active Play resource via a 3-minute video, or control parents were sent a link to a parenting website—the Raising Children Network").

1b-iii) Open vs. closed, web-based (self-assessment) vs. face-to-face assessments in the METHODS section of the ABSTRACT

Mention how participants were recruited (online vs. offline), e.g., from an open access website or from a clinic or a closed online user group (closed usergroup trial), and clarify if this was a purely web-based trial, or there were face-to-face components (as part of the intervention or for assessment). Clearly say if outcomes were self-assessed through questionnaires (as common in web-based trials). Note: In traditional offline trials, an open trial (open-label trial) is a type of clinical trial in which both the researchers and participants know which treatment is being administered. To avoid confusion, use “blinded” or “unblinded” to indicated the level of blinding instead of “open”, as “open” in web-based trials usually refers to “open access” (i.e. participants can self-enrol). (Note: Only report in the abstract what the main paper is reporting. If this information is missing from the main body of text, consider adding it)

	1	2	3	4	5	
subitem not at all important	○	○	○	○	○	essential

Does your paper address subitem 1b-iii?

Copy and paste relevant sections from the manuscript abstract (include quotes in quotation marks "like this" to indicate direct quotes from your manuscript), or elaborate on this item by providing additional information not in the ms, or briefly explain why the item is not applicable/relevant for your study

We mention in the Methods section of the abstract that participants were recruited online, and clarify that the resources that were received were online resources, and that control participants received a link to a website ("A pilot randomised controlled trial design recruited online 184 Australian parents of children 2-4 years... If they reported any parent or child negative affect or emotion dysregulation, they were randomised in-the-moment to receive an online Emotion Coaching or Active Play resource via a 3-minute video, or control parents were sent a link to a parenting website—the Raising Children Network"). It is clear that outcomes were self-assessed through questionnaires, as we mention that participants complete an online baseline- and post-survey, as well as online EMI surveys received via email ("Participants completed a 25-minute online survey at baseline and two-weeks’ post. Over two weeks, participants received twice-daily 1-minute EMI surveys via email").

1b-iv) RESULTS section in abstract must contain use data

Report number of participants enrolled/assessed in each group, the use/uptake of the intervention (e.g., attrition/adherence metrics, use over time, number of logins etc.), in addition to primary/secondary outcomes. (Note: Only report in the abstract what the main paper is reporting. If this information is missing from the main body of text, consider adding it)

	1	2	3	4	5	
subitem not at all important	○	○	○	○	○	essential

Does your paper address subitem 1b-iv?

Copy and paste relevant sections from the manuscript abstract (include quotes in quotation marks "like this" to indicate direct quotes from your manuscript), or elaborate on this item by providing additional information not in the ms, or briefly explain why the item is not applicable/relevant for your study

We report the number of participants recruited for each group within the Methods section of the Abstract ("A pilot randomised controlled trial design recruited online 184 Australian parents of children 2-4 years and randomised to intervention (n=96) or active control (n=88)"). Use and uptake are reported within the Results section of the Abstract, as we state that "70% were retained to 2-week post survey, and two-thirds completed at least half of the daily surveys". Results for acceptability as an additional primary outcome to feasibility are also stated (e.g., "most parents reported that Daily Growth was easy to use (77%)").

1b-v) CONCLUSIONS/DISCUSSION in abstract for negative trials

Conclusions/Discussions in abstract for negative trials: Discuss the primary outcome - if the trial is negative (primary outcome not changed), and the intervention was not used, discuss whether negative results are attributable to lack of uptake and discuss reasons. (Note: Only report in the abstract what the main paper is reporting. If this information is missing from the main body of text, consider adding it)

	1	2	3	4	5	
subitem not at all important	○	○	○	○	○	essential

Does your paper address subitem 1b-v?

Copy and paste relevant sections from the manuscript abstract (include quotes in quotation marks "like this" to indicate direct quotes from your manuscript), or elaborate on this item by providing additional information not in the ms, or briefly explain why the item is not applicable/relevant for your study

Our primary outcomes were feasibility and acceptability, so this is not applicable.

INTRODUCTION

2a) In INTRODUCTION: Scientific background and explanation of rationale

2a-i) Problem and the type of system/solution

Describe the problem and the type of system/solution that is object of the study: intended as stand-alone intervention vs. incorporated in broader health care program? Intended for a particular patient population? Goals of the intervention, e.g., being more cost-effective to other interventions, replace or complement other solutions? (Note: Details about the intervention are provided in “Methods” under 5)

	1	2	3	4	5	
subitem not at all important	○	○	○	○	○	essential

Does your paper address subitem 2a-i? *

Copy and paste relevant sections from the manuscript (include quotes in quotation marks "like this" to indicate direct quotes from your manuscript), or elaborate on this item by providing additional information not in the ms, or briefly explain why the item is not applicable/relevant for your study

We state in the manuscript that Daily Growth is a stand-alone parenting app ("This study is part of a larger research project developing Daily Growth, a personalised stand-alone parenting app for parents of children aged 2–4 years"). We clearly describe the gaps in the research regarding low reach and engagement of existing interventions ("the reach of programs has been extremely low (i.e., less than 10% of the general population), particularly for First Nations/Indigenous families, linguistically and culturally diverse families, fathers, and single parents"). We state that the program is a universal program for all Australian parents with a child aged 2-5 ("To address barriers to reach, access, and engagement in population-level parenting programs"). We state the goals of the program ("Daily Growth aims to enhance parent emotion regulation and parenting skills, ultimately supporting children's emotion regulation and reducing rates of child mental illness. To address barriers to reach, access, and engagement in population-level parenting programs...").

2a-ii) Scientific background, rationale: What is known about the (type of) system

Scientific background, rationale: What is known about the (type of) system that is the object of the study (be sure to discuss the use of similar systems for other conditions/diagnoses, if appropiate), motivation for the study, i.e. what are the reasons for and what is the context for this specific study, from which stakeholder viewpoint is the study performed, potential impact of findings [2]. Briefly justify the choice of the comparator.

	1	2	3	4	5	
subitem not at all important	○	○	○	○	○	essential

Does your paper address subitem 2a-ii? *

Copy and paste relevant sections from the manuscript (include quotes in quotation marks "like this" to indicate direct quotes from your manuscript), or elaborate on this item by providing additional information not in the ms, or briefly explain why the item is not applicable/relevant for your study

We describe existing parenting programs and their limitations ("A range of parenting interventions or prevention programs have been developed to decrease the burden of child mental health problems"), which our programs aims to address ("To address barriers to reach, access, and engagement in population-level parenting programs, the platform integrates novel technology approaches successfully applied in adult health settings"). We also describe the implication of our findings ("The current pilot study forms part of this phase and aims to evaluate the feasibility and acceptability of the EMI survey delivery method and a small-scale prototype of the Daily Growth program through a randomised controlled trial"). We justify our choice of comparator ("The Raising Children Network provides high-quality, but general parenting information, and does not include targeted intervention strategies as delivered via the Daily Growth program resources. Therefore, it was deemed an appropriate comparator to evaluate program efficacy").

2b) In INTRODUCTION: Specific objectives or hypotheses

Does your paper address CONSORT subitem 2b? *

Copy and paste relevant sections from the manuscript (include quotes in quotation marks "like this" to indicate direct quotes from your manuscript), or elaborate on this item by providing additional information not in the ms, or briefly explain why the item is not applicable/relevant for your study

We clearly state our aims at the end of the introduction ("Specifically, we aimed to assess:...").

METHODS

3a) Description of trial design (such as parallel, factorial) including allocation ratio

Does your paper address CONSORT subitem 3a? *

Copy and paste relevant sections from the manuscript (include quotes in quotation marks "like this" to indicate direct quotes from your manuscript), or elaborate on this item by providing additional information not in the ms, or briefly explain why the item is not applicable/relevant for your study

The trial design is described within the 'Research Design and Procedure' section, including allocation ratio ("On completion, they were randomly assigned to either the control or intervention group using a 1:1 ratio generator"). We also describe the in-the-moment randomisation of the 2 different video resources ("Intervention participants were randomised to receive either an Emotion Coaching or Active Play 3-minute video resource").

3b) Important changes to methods after trial commencement (such as eligibility criteria), with reasons

Does your paper address CONSORT subitem 3b? *

Copy and paste relevant sections from the manuscript (include quotes in quotation marks "like this" to indicate direct quotes from your manuscript), or elaborate on this item by providing additional information not in the ms, or briefly explain why the item is not applicable/relevant for your study

We did make changes to the methods after trial commencement, which we describe in the 'Research Design and Procedure' section ("The post-EMI survey was initially programmed to be sent only if participants had completed their pre-EMI survey within 15 minutes of receiving the invitation email. As a result, participants were not always sent the post-EMI survey. This issue was identified and corrected midway through the trial by removing the time restriction...").

3b-i) Bug fixes, Downtimes, Content Changes

Bug fixes, Downtimes, Content Changes: ehealth systems are often dynamic systems. A description of changes to methods therefore also includes important changes made on the intervention or comparator during the trial (e.g., major bug fixes or changes in the functionality or content) (5-iii) and other “unexpected events” that may have influenced study design such as staff changes, system failures/downtimes, etc. [2].

	1	2	3	4	5	
subitem not at all important	○	○	○	○	○	essential

Does your paper address subitem 3b-i?

Copy and paste relevant sections from the manuscript (include quotes in quotation marks "like this" to indicate direct quotes from your manuscript), or elaborate on this item by providing additional information not in the ms, or briefly explain why the item is not applicable/relevant for your study

We did not make any changes to the program resources themselves after beginning the trial.

4a) Eligibility criteria for participants

Does your paper address CONSORT subitem 4a? *

Copy and paste relevant sections from the manuscript (include quotes in quotation marks "like this" to indicate direct quotes from your manuscript), or elaborate on this item by providing additional information not in the ms, or briefly explain why the item is not applicable/relevant for your study

We do clearly state eligibility criteria ("Participants were eligible if they were residing in Australia, 18 years or older, and a current parent or carer (i.e., biological, step, co-parent) of a child aged 2 to 4 years").

4a-i) Computer / Internet literacy

Computer / Internet literacy is often an implicit “de facto” eligibility criterion - this should be explicitly clarified.

	1	2	3	4	5	
subitem not at all important	○	○	○	○	○	essential

Does your paper address subitem 4a-i?

Copy and paste relevant sections from the manuscript (include quotes in quotation marks "like this" to indicate direct quotes from your manuscript), or elaborate on this item by providing additional information not in the ms, or briefly explain why the item is not applicable/relevant for your study

We clarify that participants need to have basic computer/internet literacy skills ("Participants also needed to be able to understand English, have access to an internet-enabled device, and have basic digital literacy skills (such as being able to receive emails and filling out online surveys)").

4a-ii) Open vs. closed, web-based vs. face-to-face assessments:

Open vs. closed, web-based vs. face-to-face assessments: Mention how participants were recruited (online vs. offline), e.g., from an open access website or from a clinic, and clarify if this was a purely web-based trial, or there were face-to-face components (as part of the intervention or for assessment), i.e., to what degree got the study team to know the participant. In online-only trials, clarify if participants were quasi-anonymous and whether having multiple identities was possible or whether technical or logistical measures (e.g., cookies, email confirmation, phone calls) were used to detect/prevent these.

	1	2	3	4	5	
subitem not at all important	○	○	○	○	○	essential

Does your paper address subitem 4a-ii? *

Copy and paste relevant sections from the manuscript (include quotes in quotation marks "like this" to indicate direct quotes from your manuscript), or elaborate on this item by providing additional information not in the ms, or briefly explain why the item is not applicable/relevant for your study

We describe our recruitment process in the section 'Participants and Recruitment' ("We contacted parents who previously participated in similar research, posted unpaid advertisements on Facebook, and paid advertisements using the Meta ad manager platform for Facebook and Instagram"). We describe asessements and delivery of the resources within the section 'Research Design and Procedure' ("Participants registered to the web platform 'Platform O' and completed a 25-minute baseline survey via Qualtrics...", "Over 14 days, participants received automated twice-daily 1-minute EMI survey prompts via email at 7.30am and 7.30pm", "Intervention participants were randomised to receive either an Emotion Coaching or Active Play 3-minute video resource"), making clear that all components are delivered online. We also describe our method of checking for duplicate participants ("Duplicate participants were identified by cross-checking email addresses").

4a-iii) Information giving during recruitment

Information given during recruitment. Specify how participants were briefed for recruitment and in the informed consent procedures (e.g., publish the informed consent documentation as appendix, see also item X26), as this information may have an effect on user self-selection, user expectation and may also bias results.

	1	2	3	4	5	
subitem not at all important	○	○	○	○	○	essential

Does your paper address subitem 4a-iii?

Copy and paste relevant sections from the manuscript (include quotes in quotation marks "like this" to indicate direct quotes from your manuscript), or elaborate on this item by providing additional information not in the ms, or briefly explain why the item is not applicable/relevant for your study

We submit the PLS and online consent form as an appendix. We also explain our approach for informed consent in the Methods section ("Participants provided online with informed consent via a checkbox before participation. Participants indicated whether they consent to participate in the study and to be contacted about future research. Participants received a Plain Language Statement (PLS) outlining the study procedures and purpose, estimated duration of each study component, their right to withdraw at any time, the policy regarding data storage, and contact details for the Principal Investigator (EW) for questions or concerns").

4b) Settings and locations where the data were collected

Does your paper address CONSORT subitem 4b? *

Copy and paste relevant sections from the manuscript (include quotes in quotation marks "like this" to indicate direct quotes from your manuscript), or elaborate on this item by providing additional information not in the ms, or briefly explain why the item is not applicable/relevant for your study

Yes, we state that the trial was completely online, and that all parents (with a child in the right age range) could enrol as long as they are located in Australia ("Participants were eligible if they were residing in Australia...", "Participants registered to the web platform 'Platform O' and completed a 25-minute baseline survey via Qualtrics.... participants received automated twice-daily 1-minute EMI survey prompts via email").

4b-i) Report if outcomes were (self-)assessed through online questionnaires

Clearly report if outcomes were (self-)assessed through online questionnaires (as common in web-based trials) or otherwise.

	1	2	3	4	5	
subitem not at all important	○	○	○	○	○	essential

Does your paper address subitem 4b-i? *

Copy and paste relevant sections from the manuscript (include quotes in quotation marks "like this" to indicate direct quotes from your manuscript), or elaborate on this item by providing additional information not in the ms, or briefly explain why the item is not applicable/relevant for your study

Yes, we describe all of our outcome measures, and state that they were administered online via Qualtrics ("Participants registered to the web platform 'Platform O' and completed a 25-minute baseline survey via Qualtrics... "At the end of the 2-week parenting program period, participants were invited to complete a 25-minute post-intervention survey via Qualtrics").

4b-ii) Report how institutional affiliations are displayed

Report how institutional affiliations are displayed to potential participants [on ehealth media], as affiliations with prestigious hospitals or universities may affect volunteer rates, use, and reactions with regards to an intervention.(Not a required item – describe only if this may bias results)

	1	2	3	4	5	
subitem not at all important	○	○	○	○	○	essential

Does your paper address subitem 4b-ii?

Copy and paste relevant sections from the manuscript (include quotes in quotation marks "like this" to indicate direct quotes from your manuscript), or elaborate on this item by providing additional information not in the ms, or briefly explain why the item is not applicable/relevant for your study

We did not report on how institutional affiliations are displayed to participants - we acknowledge the possibility that participants may be more inclined to engage in a program if it stems from a prestigious institution, however we do not believe that our program's affiliation with Deakin University skewed the outcomes of this study.

5) The interventions for each group with sufficient details to allow replication, including how and when they were actually administered

5-i) Mention names, credential, affiliations of the developers, sponsors, and owners

Mention names, credential, affiliations of the developers, sponsors, and owners [6] (if authors/evaluators are owners or developer of the software, this needs to be declared in a “Conflict of interest” section or mentioned elsewhere in the manuscript).

	1	2	3	4	5	
subitem not at all important	◯	◯	◯	◯	◯	essential

Does your paper address subitem 5-i?

Copy and paste relevant sections from the manuscript (include quotes in quotation marks "like this" to indicate direct quotes from your manuscript), or elaborate on this item by providing additional information not in the ms, or briefly explain why the item is not applicable/relevant for your study

Names, credentials, and affiliations of the developers are stated in the Methods section ("The Daily Growth parenting program was developed by A/Prof Elizabeth Westrupp, Mrs Maria Bates, Ms Kayla Redpath, Mr Eric O, Dr Kelsie Bufton, Ms Stefanie Ewald, Mr Storm Hiskens-Ravest, Mr Tomer Berkowitz, and Dr Gabriella King at Deakin University, Australia").

5-ii) Describe the history/development process

Describe the history/development process of the application and previous formative evaluations (e.g., focus groups, usability testing), as these will have an impact on adoption/use rates and help with interpreting results.

	1	2	3	4	5	
subitem not at all important	◯	◯	◯	◯	◯	essential

Does your paper address subitem 5-ii?

Copy and paste relevant sections from the manuscript (include quotes in quotation marks "like this" to indicate direct quotes from your manuscript), or elaborate on this item by providing additional information not in the ms, or briefly explain why the item is not applicable/relevant for your study

We describe the co-design process of the program and initial testing in the Introduction, in the section 'The Current Study: Daily Growth' ("In the Understand phase, a needs assessment was undertaken through a review of relevant literature and 17 one-hour interviews with parents, alongside four one-hour interviews with professionals", "The Co-develop phase involved collaborating with users and stakeholders to develop the program's user journey and content", "Additionally, an ecological momentary assessment study involving 89 parents/carers was conducted to inform the development of an appropriate protocol for delivering daily surveys").

5-iii) Revisions and updating

Revisions and updating. Clearly mention the date and/or version number of the application/intervention (and comparator, if applicable) evaluated, or describe whether the intervention underwent major changes during the evaluation process, or whether the development and/or content was "frozen" during the trial. Describe dynamic components such as news feeds or changing content which may have an impact on the replicability of the intervention (for unexpected events see item 3b).

	1	2	3	4	5	
subitem not at all important	○	○	○	○	○	essential

Does your paper address subitem 5-iii?

Copy and paste relevant sections from the manuscript (include quotes in quotation marks "like this" to indicate direct quotes from your manuscript), or elaborate on this item by providing additional information not in the ms, or briefly explain why the item is not applicable/relevant for your study

We state that this program is a prototype version ("The current pilot evaluated a prototype version of Daily Growth") and we describe that while program content was frozen during the trial, the program has since been further developed into an app ("Program content was frozen throughout the trial, however changes were made regarding the EMI-survey delivery approach", "The final Daily Growth app will feature 90+ video resources delivered to parents over six weeks via a custom-built smartphone app for iOS and Android").

5-iv) Quality assurance methods

Provide information on quality assurance methods to ensure accuracy and quality of information provided [1], if applicable.

	1	2	3	4	5	
subitem not at all important	○	○	○	○	○	essential

Does your paper address subitem 5-iv?

Copy and paste relevant sections from the manuscript (include quotes in quotation marks "like this" to indicate direct quotes from your manuscript), or elaborate on this item by providing additional information not in the ms, or briefly explain why the item is not applicable/relevant for your study

We did not employ any quality assurance methods.

5-v) Ensure replicability by publishing the source code, and/or providing screenshots/screen-capture video, and/or providing flowcharts of the algorithms used

Ensure replicability by publishing the source code, and/or providing screenshots/screen-capture video, and/or providing flowcharts of the algorithms used. Replicability (i.e., other researchers should in principle be able to replicate the study) is a hallmark of scientific reporting.

	1	2	3	4	5	
subitem not at all important	○	○	○	○	○	essential

Does your paper address subitem 5-v?

Copy and paste relevant sections from the manuscript (include quotes in quotation marks "like this" to indicate direct quotes from your manuscript), or elaborate on this item by providing additional information not in the ms, or briefly explain why the item is not applicable/relevant for your study

Since the program has since been developed into an app and the current pilot study tested a prototype version of the program, we do not have access to the source code. However, we clearly describe program design and components in the Methods section.

5-vi) Digital preservation

Digital preservation: Provide the URL of the application, but as the intervention is likely to change or disappear over the course of the years; also make sure the intervention is archived (Internet Archive, webcitation.org, and/or publishing the source code or screenshots/videos alongside the article). As pages behind login screens cannot be archived, consider creating demo pages which are accessible without login.

	1	2	3	4	5	
subitem not at all important	○	○	○	○	○	essential

Does your paper address subitem 5-vi?

Copy and paste relevant sections from the manuscript (include quotes in quotation marks "like this" to indicate direct quotes from your manuscript), or elaborate on this item by providing additional information not in the ms, or briefly explain why the item is not applicable/relevant for your study

Since the prototype version of the program has since been developed into an app which is currently accessible via the Apple/Google Play Store, the URL of the prototype version is not accessible anymore.

5-vii) Access

Access: Describe how participants accessed the application, in what setting/context, if they had to pay (or were paid) or not, whether they had to be a member of specific group. If known, describe how participants obtained “access to the platform and Internet” [1]. To ensure access for editors/reviewers/readers, consider to provide a “backdoor” login account or demo mode for reviewers/readers to explore the application (also important for archiving purposes, see vi).

	1	2	3	4	5	
subitem not at all important	○	○	○	○	○	essential

Does your paper address subitem 5-vii? *

Copy and paste relevant sections from the manuscript (include quotes in quotation marks "like this" to indicate direct quotes from your manuscript), or elaborate on this item by providing additional information not in the ms, or briefly explain why the item is not applicable/relevant for your study

In the section 'Research Design and Procedure' we describe how participants accessed the program ("Participants registered to the web platform ‘Platform O’ and completed a 25-minute baseline survey via Qualtrics", "participants received automated twice-daily 1-minute EMI survey prompts via email... Intervention participants were randomised to receive either an Emotion Coaching or Active Play 3-minute video resource"). We also explain the inclusion criteria and the reimbursement approach ("Parents were reimbursed for their contribution. Each participant’s gift voucher reflected the percentage of prompts they completed (50-69%=$20; 70-84%=$30; 85-100%=$50)").The program has since been moved from the online platform to an app format, so unfortunately we cannot provide a "backdoor" login account.

5-viii) Mode of delivery, features/functionalities/components of the intervention and comparator, and the theoretical framework

Describe mode of delivery, features/functionalities/components of the intervention and comparator, and the theoretical framework [6] used to design them (instructional strategy [1], behaviour change techniques, persuasive features, etc., see e.g., [7, 8] for terminology). This includes an in-depth description of the content (including where it is coming from and who developed it) [1]," whether [and how] it is tailored to individual circumstances and allows users to track their progress and receive feedback" [6]. This also includes a description of communication delivery channels and – if computer-mediated communication is a component – whether communication was synchronous or asynchronous [6]. It also includes information on presentation strategies [1], including page design principles, average amount of text on pages, presence of hyperlinks to other resources, etc. [1].

	1	2	3	4	5	
subitem not at all important	○	○	○	○	○	essential

Does your paper address subitem 5-viii? *

Copy and paste relevant sections from the manuscript (include quotes in quotation marks "like this" to indicate direct quotes from your manuscript), or elaborate on this item by providing additional information not in the ms, or briefly explain why the item is not applicable/relevant for your study

We describe the mode of delivery and the active control group component in the section 'Research Design and Procedure' ("Participants registered to the web platform 'Platform O' and completed a 25-minute baseline survey via Qualtrics", "Over 14 days, participants received automated twice-daily 1-minute EMI survey prompts via email", "Control participants were sent a link to the Raising Children Network government website, tailored to their selected parenting situation"). We describe the program components in detail including the theoretical framework, and the communication delivery channel (video resource) in the section 'Daily Growth Program Resources' ("The Daily Growth parenting program provides in-the-moment support using a structured emotion socialization approach", "The Emotion Coaching resources are based on Gottman et al.'s five essential steps of emotion coaching, and help parents become more aware of their own emotions and view their child's feelings as opportunities for connection and learning...", "The Active Play resources help parents use movement and play-based activities to navigate parenting situations, such as winding down before bedtime..."). We do not track 'progress' or give parents 'feedback', as this program is designed to provide parents with support that they can adapt to their own lives to suit their needs. The program does include a tailoring component however, as parents may select a difficult parenting situation they recently experienced, and receive a resource tailored to this specific situation ("They were then asked to select one of five difficult parenting situations they had recently experienced. Control participants were sent a link to the Raising Children Network government website, tailored to their selected parenting situation. Intervention participants were randomised to receive either an Emotion Coaching or Active Play 3-minute video resource tailored to their selected parenting situation"). We describe presentation of the resources in detail in the section 'Daily Growth Program Resources' ("Each three-minute video resource uses animation, live footage of real parents and children, and expert 'talking heads' speaking with subtitles").

5-ix) Describe use parameters

Describe use parameters (e.g., intended "doses" and optimal timing for use). Clarify what instructions or recommendations were given to the user, e.g., regarding timing, frequency, heaviness of use, if any, or was the intervention used ad libitum.

	1	2	3	4	5	
subitem not at all important	○	○	○	○	○	essential

Does your paper address subitem 5-ix?

Copy and paste relevant sections from the manuscript (include quotes in quotation marks "like this" to indicate direct quotes from your manuscript), or elaborate on this item by providing additional information not in the ms, or briefly explain why the item is not applicable/relevant for your study

We do clearly describe use parameters, including when survey prompts were sent out ("Over 14 days, participants received automated twice-daily 1-minute EMI survey prompts via email at 7.30am and 7.30pm (the pre-EMI survey)", "Finally, both conditions were prompted via email to complete a post-EMI survey at 7:45am and 7:45pm"). Participants were also aware of how much they would need to engage with the program to receive reimbursement ("Each participant’s gift voucher reflected the percentage of prompts they completed (50-69%=$20; 70-84%=$30; 85-100%=$50)").

5-x) Clarify the level of human involvement

Clarify the level of human involvement (care providers or health professionals, also technical assistance) in the e-intervention or as co-intervention (detail number and expertise of professionals involved, if any, as well as “type of assistance offered, the timing and frequency of the support, how it is initiated, and the medium by which the assistance is delivered”. It may be necessary to distinguish between the level of human involvement required for the trial, and the level of human involvement required for a routine application outside of a RCT setting (discuss under item 21 – generalizability).

	1	2	3	4	5	
subitem not at all important	○	○	○	○	○	essential

Does your paper address subitem 5-x?

Copy and paste relevant sections from the manuscript (include quotes in quotation marks "like this" to indicate direct quotes from your manuscript), or elaborate on this item by providing additional information not in the ms, or briefly explain why the item is not applicable/relevant for your study

Our program was completely online, so this is not applicable.

5-xi) Report any prompts/reminders used

Report any prompts/reminders used: Clarify if there were prompts (letters, emails, phone calls, SMS) to use the application, what triggered them, frequency etc. It may be necessary to distinguish between the level of prompts/reminders required for the trial, and the level of prompts/reminders for a routine application outside of a RCT setting (discuss under item 21 – generalizability).

	1	2	3	4	5	
subitem not at all important	○	○	○	○	○	essential

Does your paper address subitem 5-xi? *

Copy and paste relevant sections from the manuscript (include quotes in quotation marks "like this" to indicate direct quotes from your manuscript), or elaborate on this item by providing additional information not in the ms, or briefly explain why the item is not applicable/relevant for your study

We used EMI survey prompts which were sent to participants twice daily ("Over 14 days, participants received automated twice-daily 1-minute EMI survey prompts via email at 7.30am and 7.30pm (the pre-EMI survey)", "Finally, both conditions were prompted via email to complete a post-EMI survey at 7:45am and 7:45pm").

5-xii) Describe any co-interventions (incl. training/support)

Describe any co-interventions (incl. training/support): Clearly state any interventions that are provided in addition to the targeted eHealth intervention, as ehealth intervention may not be designed as stand-alone intervention. This includes training sessions and support [1]. It may be necessary to distinguish between the level of training required for the trial, and the level of training for a routine application outside of a RCT setting (discuss under item 21 – generalizability.

	1	2	3	4	5	
subitem not at all important	○	○	○	○	○	essential

Does your paper address subitem 5-xii? *

Copy and paste relevant sections from the manuscript (include quotes in quotation marks "like this" to indicate direct quotes from your manuscript), or elaborate on this item by providing additional information not in the ms, or briefly explain why the item is not applicable/relevant for your study

This program does not include any co-interventions, so this is not applicable.

6a) Completely defined pre-specified primary and secondary outcome measures, including how and when they were assessed

Does your paper address CONSORT subitem 6a? *

Copy and paste relevant sections from the manuscript (include quotes in quotation marks "like this" to indicate direct quotes from your manuscript), or elaborate on this item by providing additional information not in the ms, or briefly explain why the item is not applicable/relevant for your study

We describe both primary and secondary outcome measures, including how and when they were assessed ("Recruitment was evaluated based on the percentage of eligible participants who completed the baseline survey after clicking on the study link, and the total number of parents enrolled", "Retention was measured by attrition rates, tracking participant drop-out and withdrawal between baseline and post-intervention surveys over the 2-week study period", "Engagement was measured by determining the proportion of completed prompts per parent, the timing of responses, and parent-reported barriers to participation", "A user satisfaction survey was created to assess the acceptability of the Daily Growth resources... The measure was administered to parents at the end of the 2-week parenting program as part of the 25-minute post-survey", "The baseline and post-intervention surveys assessed parent and child emotion regulation, parent and child mental health, emotion coaching parenting practices, and sociodemographic information. The twice-daily EMI pre- and post-surveys were identical, and parent and child emotion dysregulation and negative affect were evaluated in-the-moment", "Participants registered to the web platform ‘Platform O’ and completed a 25-minute baseline survey via Qualtrics", "Over 14 days, participants received automated twice-daily 1-minute EMI survey prompts via email at 7.30am and 7.30pm (the pre-EMI survey)", "Finally, both conditions were prompted via email to complete a post-EMI survey at 7:45am and 7:45pm", "At the end of the 2-week parenting program period, participants were invited to complete a 25-minute post-intervention survey via Qualtrics").

6a-i) Online questionnaires: describe if they were validated for online use and apply CHERRIES items to describe how the questionnaires were designed/deployed

If outcomes were obtained through online questionnaires, describe if they were validated for online use and apply CHERRIES items to describe how the questionnaires were designed/deployed [9].

	1	2	3	4	5	
subitem not at all important	○	○	○	○	○	essential

Does your paper address subitem 6a-i?

Copy and paste relevant sections from manuscript text

The user satisfaction questionnaire was created specifically for this study so had not been validated beforehand, however, this pilot study aimed to test the feasibility of the methods so they can be adjusted for the larger Daily Growth trial, which is why validation beforehand was not necessary in this case. The EMI surveys were previously validated as part of an EMA study ("To identify the most suitable state-based items for assessing in-the-moment parent and child emotion dysregulation and negative affect, an ecological momentary assessment study was conducted in 2022"). The baseline and post-survey consist of well-known, previously validates measures, including the MAP-DB, SMFQ, SCAS, EERBQ, PANAS, DERS, K6, DASS, CTNES-SF, PRFQ, PBACE, and SEFQ. CHERRIES items (e.g. number of items/pages, completeness checks) are included in the Methods section ("The baseline survey included 424 items across 100 pages. No completeness checks were included in the Qualtrics surveys, however...").

6a-ii) Describe whether and how “use” (including intensity of use/dosage) was defined/measured/monitored

Describe whether and how “use” (including intensity of use/dosage) was defined/measured/monitored (logins, logfile analysis, etc.). Use/adoption metrics are important process outcomes that should be reported in any ehealth trial.

	1	2	3	4	5	
subitem not at all important	○	○	○	○	○	essential

Does your paper address subitem 6a-ii?

Copy and paste relevant sections from manuscript text

Use was monitored since engagement was one of our primary outcomes. We measured the amount of times participants responded to survey prompts ("Engagement was measured by determining the proportion of completed prompts per parent, the timing of responses, ...").

6a-iii) Describe whether, how, and when qualitative feedback from participants was obtained

Describe whether, how, and when qualitative feedback from participants was obtained (e.g., through emails, feedback forms, interviews, focus groups).

	1	2	3	4	5	
subitem not at all important	○	○	○	○	○	essential

Does your paper address subitem 6a-iii?

Copy and paste relevant sections from manuscript text

Participants could submit qualitative feedback via the user satisfaction questionnaire ("A user satisfaction survey was created to assess the acceptability of the Daily Growth resources. The survey consisted of 25 items, combining 5-point Likert scale items (e.g., "The Daily Growth program meets my needs."), multiple-choice questions (e.g., "Which parenting resource did you prefer?"), and open-ended questions (e.g., "What problems did you encounter while using the program?")").

6b) Any changes to trial outcomes after the trial commenced, with reasons

Does your paper address CONSORT subitem 6b? *

Copy and paste relevant sections from the manuscript (include quotes in quotation marks "like this" to indicate direct quotes from your manuscript), or elaborate on this item by providing additional information not in the ms, or briefly explain why the item is not applicable/relevant for your study

No, trial outcomes were not changed after the trial commenced. However, we did change the delivery of the post-EMI survey (not the content): "The post-EMI survey was initially programmed to be sent only if participants had completed their pre-EMI survey within 15 minutes of receiving the invitation email. As a result, participants were not always sent the post-EMI survey. This issue was identified and corrected midway through the trial by removing the time restriction. Additionally, the post-EMI survey prompt was changed to be sent to all participants, regardless of their pre-EMI survey score."

7a) How sample size was determined

NPT: When applicable, details of whether and how the clustering by care provides or centers was addressed

7a-i) Describe whether and how expected attrition was taken into account when calculating the sample size

Describe whether and how expected attrition was taken into account when calculating the sample size.

	1	2	3	4	5	
subitem not at all important	○	○	○	○	○	essential

Does your paper address subitem 7a-i?

Copy and paste relevant sections from manuscript title (include quotes in quotation marks "like this" to indicate direct quotes from your manuscript), or elaborate on this item by providing additional information not in the ms, or briefly explain why the item is not applicable/relevant for your study

This is not applicable to our study, as this was a pilot RCT and our intention was to test feasibility rather than efficacy, so we did not conduct a power calculation.

7b) When applicable, explanation of any interim analyses and stopping guidelines

Does your paper address CONSORT subitem 7b? *

Copy and paste relevant sections from the manuscript (include quotes in quotation marks "like this" to indicate direct quotes from your manuscript), or elaborate on this item by providing additional information not in the ms, or briefly explain why the item is not applicable/relevant for your study

We did not conduct interim analyses and did not implement formal stopping guidelines.

8a) Method used to generate the random allocation sequence

NPT: When applicable, how care providers were allocated to each trial group

Does your paper address CONSORT subitem 8a? *

Copy and paste relevant sections from the manuscript (include quotes in quotation marks "like this" to indicate direct quotes from your manuscript), or elaborate on this item by providing additional information not in the ms, or briefly explain why the item is not applicable/relevant for your study

Participants were assigned using an automatic generator ("On completion, they were randomly assigned to either the control or intervention group using a 1:1 ratio generator. Randomisation occurred when participants commenced the baseline survey and was computer generated using a pseudo-random number generator on the survey platform Qualtrics").

8b) Type of randomisation; details of any restriction (such as blocking and block size)

Does your paper address CONSORT subitem 8b? *

Copy and paste relevant sections from the manuscript (include quotes in quotation marks "like this" to indicate direct quotes from your manuscript), or elaborate on this item by providing additional information not in the ms, or briefly explain why the item is not applicable/relevant for your study

We describe the type of randomisation in the Methods section ("On completion, they were randomly assigned to either the control or intervention group using a 1:1 ratio generator"). However, we did not implement any restrictions.

9) Mechanism used to implement the random allocation sequence (such as sequentially numbered containers), describing any steps taken to conceal the sequence until interventions were assigned

Does your paper address CONSORT subitem 9? *

Copy and paste relevant sections from the manuscript (include quotes in quotation marks "like this" to indicate direct quotes from your manuscript), or elaborate on this item by providing additional information not in the ms, or briefly explain why the item is not applicable/relevant for your study

We describe the randomisation mechanism in detail in the Methods section ("Randomisation occurred when participants commenced the baseline survey and was computer generated using a pseudo-random number generator on the survey platform Qualtrics. All participants were blinded to participant allocation status, but staff were not").

10) Who generated the random allocation sequence, who enrolled participants, and who assigned participants to interventions

Does your paper address CONSORT subitem 10? *

Copy and paste relevant sections from the manuscript (include quotes in quotation marks "like this" to indicate direct quotes from your manuscript), or elaborate on this item by providing additional information not in the ms, or briefly explain why the item is not applicable/relevant for your study

The randomisation and enrolment process was automated, and randomisation to intervention vs. control was done using an automatic generator via Qualtrics (""On completion, they were randomly assigned to either the control or intervention group using a 1:1 ratio generator. Randomisation occurred when participants commenced the baseline survey and was computer generated using a pseudo-random number generator on the survey platform Qualtrics "). Participants enrolled themselves by registering to Platform O ("Participants registered to the web platform 'Platform O' and completed a 25-minute baseline survey via Qualtrics"). Intervention participants were automatically randomised again in-the-moment to receive either one of the two program types ("Intervention participants were randomised to receive either an Emotion Coaching or Active Play 3-minute video resource tailored to their selected parenting situation").

11a) If done, who was blinded after assignment to interventions (for example, participants, care providers, those assessing outcomes) and how

NPT: Whether or not administering co-interventions were blinded to group assignment

11a-i) Specify who was blinded, and who wasn't

Specify who was blinded, and who wasn't. Usually, in web-based trials it is not possible to blind the participants [1, 3] (this should be clearly acknowledged), but it may be possible to blind outcome assessors, those doing data analysis or those administering co-interventions (if any).

	1	2	3	4	5	
subitem not at all important	○	○	○	○	○	essential

Does your paper address subitem 11a-i? *

Copy and paste relevant sections from the manuscript (include quotes in quotation marks "like this" to indicate direct quotes from your manuscript), or elaborate on this item by providing additional information not in the ms, or briefly explain why the item is not applicable/relevant for your study

Participants were blinded, as they did not know whether they were in the control or in the intervention group. However, participants in the intervention group were aware that they would be receiving two types of interventions. Outcome assessors or data analysts were not blinded.

11a-ii) Discuss e.g., whether participants knew which intervention was the "intervention of interest" and which one was the "comparator"

Informed consent procedures (4a-ii) can create biases and certain expectations - discuss e.g., whether participants knew which intervention was the "intervention of interest" and which one was the "comparator".

	1	2	3	4	5	
subitem not at all important	○	○	○	○	○	essential

Does your paper address subitem 11a-ii?

Copy and paste relevant sections from the manuscript (include quotes in quotation marks "like this" to indicate direct quotes from your manuscript), or elaborate on this item by providing additional information not in the ms, or briefly explain why the item is not applicable/relevant for your study

Participants did not know which intervention was the "intervention of interest", as they were informed via the PLS that they would receive a "link to an online parenting resource". We also discuss in the Methods section that participants were unaware of their allocation status ("All participants were blinded to participant allocation status").

11b) If relevant, description of the similarity of interventions

(this item is usually not relevant for ehealth trials as it refers to similarity of a placebo or sham intervention to a active medication/intervention)

Does your paper address CONSORT subitem 11b? *

Copy and paste relevant sections from the manuscript (include quotes in quotation marks "like this" to indicate direct quotes from your manuscript), or elaborate on this item by providing additional information not in the ms, or briefly explain why the item is not applicable/relevant for your study

We clearly describe the program types and the control condition in the Methods section ("Control participants were sent a link to the Raising Children Network government website, tailored to their selected parenting situation. Intervention participants were randomised to receive either an Emotion Coaching or Active Play 3-minute video resource tailored to their selected parenting situation").

12a) Statistical methods used to compare groups for primary and secondary outcomes

NPT: When applicable, details of whether and how the clustering by care providers or centers was addressed

Does your paper address CONSORT subitem 12a? *

Copy and paste relevant sections from the manuscript (include quotes in quotation marks "like this" to indicate direct quotes from your manuscript), or elaborate on this item by providing additional information not in the ms, or briefly explain why the item is not applicable/relevant for your study

Statistical methods are clearly described in the 'Statistical Analysis' section ("To evaluate feasibility in terms of participant recruitment, retention, and engagement, data from the baseline, post-surveys, and daily EMI surveys were analysed using descriptive statistics... To evaluate acceptability of the program content and delivery method, user satisfaction items from the post-survey were analysed descriptively to assess participant feedback about the Daily Growth program. To assess feasibility of the research methods in evaluating the efficacy of Daily Growth, Bonferroni-adjusted linear regression analyses were conducted for parent and child outcomes....Additionally, pre- and post-EMI daily survey data were analysed using linear mixed-effects regression models, incorporating fixed effects (to account for group and timepoint) and random effects (to adjust for individual variations over time)...").

12a-i) Imputation techniques to deal with attrition / missing values

Imputation techniques to deal with attrition / missing values: Not all participants will use the intervention/comparator as intended and attrition is typically high in ehealth trials. Specify how participants who did not use the application or dropped out from the trial were treated in the statistical analysis (a complete case analysis is strongly discouraged, and simple imputation techniques such as LOCF may also be problematic [4]).

	1	2	3	4	5	
subitem not at all important	○	○	○	○	○	essential

Does your paper address subitem 12a-i? *

Copy and paste relevant sections from the manuscript (include quotes in quotation marks "like this" to indicate direct quotes from your manuscript), or elaborate on this item by providing additional information not in the ms, or briefly explain why the item is not applicable/relevant for your study

We did not use data imputation techniques, our analyses used the maximum available data ("Analyses used the maximum available data, resulting in variation in sample size per test").

12b) Methods for additional analyses, such as subgroup analyses and adjusted analyses

Does your paper address CONSORT subitem 12b? *

Copy and paste relevant sections from the manuscript (include quotes in quotation marks "like this" to indicate direct quotes from your manuscript), or elaborate on this item by providing additional information not in the ms, or briefly explain why the item is not applicable/relevant for your study

We used "Pearson pairwise correlations to examine associations between demographic characteristics and engagement with pre-and-post-EMI surveys and the 2-week post-survey". We also used "cluster-robust variance estimation to account for repeated observations within participants, allowing assessment of the program's in-the-moment impact on EMI outcomes, including parent and child emotion dysregulation and negative affect".

X26) REB/IRB Approval and Ethical Considerations [recommended as subheading under "Methods"] (not a CONSORT item)

X26-i) Comment on ethics committee approval

	1	2	3	4	5	
subitem not at all important	○	○	○	○	○	essential

Does your paper address subitem X26-i?

Copy and paste relevant sections from the manuscript (include quotes in quotation marks "like this" to indicate direct quotes from your manuscript), or elaborate on this item by providing additional information not in the ms, or briefly explain why the item is not applicable/relevant for your study

We state that ethics approval had been given for this study ("Study procedures were approved by the Deakin University Human Ethics Advisory Group ... (HEAG-H 188_2022)").

x26-ii) Outline informed consent procedures

Outline informed consent procedures e.g., if consent was obtained offline or online (how? Checkbox, etc.?), and what information was provided (see 4a-ii). See [6] for some items to be included in informed consent documents.

	1	2	3	4	5	
subitem not at all important	○	○	○	○	○	essential

Does your paper address subitem X26-ii?

Copy and paste relevant sections from the manuscript (include quotes in quotation marks "like this" to indicate direct quotes from your manuscript), or elaborate on this item by providing additional information not in the ms, or briefly explain why the item is not applicable/relevant for your study

We state that informed consent was obtained online ("participants provided online with informed consent via a checkbox before participation "), and what information participants provided ("Participants indicated whether they consent to participate in the study and to be contacted about future research"), as well as what information was provided to participants in the PLS ("Participants received a Plain Language Statement (PLS) outlining the study procedures and purpose, estimated duration of each study component, their right to withdraw at any time, the policy regarding data storage, and contact details for the Principal Investigator (EW) for questions or concerns").

X26-iii) Safety and security procedures

Safety and security procedures, incl. privacy considerations, and any steps taken to reduce the likelihood or detection of harm (e.g., education and training, availability of a hotline)

	1	2	3	4	5	
subitem not at all important	○	○	○	○	○	essential

Does your paper address subitem X26-iii?

Copy and paste relevant sections from the manuscript (include quotes in quotation marks "like this" to indicate direct quotes from your manuscript), or elaborate on this item by providing additional information not in the ms, or briefly explain why the item is not applicable/relevant for your study

We explain that information regarding safety and security procedures was provided to participants in the PLS ("Participants received a Plain Language Statement (PLS) outlining the study procedures and purpose, estimated duration of each study component, their right to withdraw at any time, the policy regarding data storage, and contact details for the Principal Investigator (EW) for questions or concerns").

RESULTS

13a) For each group, the numbers of participants who were randomly assigned, received intended treatment, and were analysed for the primary outcome

NPT: The number of care providers or centers performing the intervention in each group and the number of patients treated by each care provider in each center

Does your paper address CONSORT subitem 13a? *

Copy and paste relevant sections from the manuscript (include quotes in quotation marks "like this" to indicate direct quotes from your manuscript), or elaborate on this item by providing additional information not in the ms, or briefly explain why the item is not applicable/relevant for your study

The number of participants who were randomly assigned, received the interventions, and were analysed is outlined in the flowchart (Figure 1).

13b) For each group, losses and exclusions after randomisation, together with reasons

Does your paper address CONSORT subitem 13b? (NOTE: Preferably, this is shown in a CONSORT flow diagram) *

Copy and paste relevant sections from the manuscript (include quotes in quotation marks "like this" to indicate direct quotes from your manuscript), or elaborate on this item by providing additional information not in the ms, or briefly explain why the item is not applicable/relevant for your study

This information is included in the flowchart as well (Figure 1).

13b-i) Attrition diagram

Strongly recommended: An attrition diagram (e.g., proportion of participants still logging in or using the intervention/comparator in each group plotted over time, similar to a survival curve) or other figures or tables demonstrating usage/dose/engagement.

	1	2	3	4	5	
subitem not at all important	○	○	○	○	○	essential

Does your paper address subitem 13b-i?

Copy and paste relevant sections from the manuscript or cite the figure number if applicable (include quotes in quotation marks "like this" to indicate direct quotes from your manuscript), or elaborate on this item by providing additional information not in the ms, or briefly explain why the item is not applicable/relevant for your study

Figure 2 and Figure 3 show the participant flow through the two-week EMI survey prompts, and average pre-and post-EMI completion rates across study timepoints.

14a) Dates defining the periods of recruitment and follow-up

Does your paper address CONSORT subitem 14a? *

Copy and paste relevant sections from the manuscript (include quotes in quotation marks "like this" to indicate direct quotes from your manuscript), or elaborate on this item by providing additional information not in the ms, or briefly explain why the item is not applicable/relevant for your study

The period of recruitment is stated in the Methods section ("Participants were recruited online in March-April 2023").

14a-i) Indicate if critical “secular events” fell into the study period

Indicate if critical “secular events” fell into the study period, e.g., significant changes in Internet resources available or “changes in computer hardware or Internet delivery resources”

	1	2	3	4	5	
subitem not at all important	○	○	○	○	○	essential

Does your paper address subitem 14a-i?

Copy and paste relevant sections from the manuscript (include quotes in quotation marks "like this" to indicate direct quotes from your manuscript), or elaborate on this item by providing additional information not in the ms, or briefly explain why the item is not applicable/relevant for your study

This was not the case, so not applicable.

14b) Why the trial ended or was stopped (early)

Does your paper address CONSORT subitem 14b? *

Copy and paste relevant sections from the manuscript (include quotes in quotation marks "like this" to indicate direct quotes from your manuscript), or elaborate on this item by providing additional information not in the ms, or briefly explain why the item is not applicable/relevant for your study

We describe why recruitment was stopped in the Methods section ("Recruitment was closed once the target was met").

15) A table showing baseline demographic and clinical characteristics for each group

NPT: When applicable, a description of care providers (case volume, qualification, expertise, etc.) and centers (volume) in each group

Does your paper address CONSORT subitem 15? *

Copy and paste relevant sections from the manuscript (include quotes in quotation marks "like this" to indicate direct quotes from your manuscript), or elaborate on this item by providing additional information not in the ms, or briefly explain why the item is not applicable/relevant for your study

Baseline demographic characteristics are shown in Table 2.

15-i) Report demographics associated with digital divide issues

In ehealth trials it is particularly important to report demographics associated with digital divide issues, such as age, education, gender, social-economic status, computer/Internet/ehealth literacy of the participants, if known.

	1	2	3	4	5	
subitem not at all important	○	○	○	○	○	essential

Does your paper address subitem 15-i? *

Copy and paste relevant sections from the manuscript (include quotes in quotation marks "like this" to indicate direct quotes from your manuscript), or elaborate on this item by providing additional information not in the ms, or briefly explain why the item is not applicable/relevant for your study

Age, level of education, gender, and SES are included in Table 2. Computer literacy was not assessed.

16) For each group, number of participants (denominator) included in each analysis and whether the analysis was by original assigned groups

16-i) Report multiple “denominators” and provide definitions

Report multiple “denominators” and provide definitions: Report N’s (and effect sizes) “across a range of study participation [and use] thresholds” [1], e.g., N exposed, N consented, N used more than x times, N used more than y weeks, N participants “used” the intervention/comparator at specific pre-defined time points of interest (in absolute and relative numbers per group). Always clearly define “use” of the intervention.

	1	2	3	4	5	
subitem not at all important	○	○	○	○	○	essential

Does your paper address subitem 16-i? *

Copy and paste relevant sections from the manuscript (include quotes in quotation marks "like this" to indicate direct quotes from your manuscript), or elaborate on this item by providing additional information not in the ms, or briefly explain why the item is not applicable/relevant for your study

Meine Antwort

16-ii) Primary analysis should be intent-to-treat

Primary analysis should be intent-to-treat, secondary analyses could include comparing only “users”, with the appropriate caveats that this is no longer a randomized sample (see 18-i).

	1	2	3	4	5	
subitem not at all important	○	○	○	○	○	essential

Does your paper address subitem 16-ii?

Copy and paste relevant sections from the manuscript (include quotes in quotation marks "like this" to indicate direct quotes from your manuscript), or elaborate on this item by providing additional information not in the ms, or briefly explain why the item is not applicable/relevant for your study

We clearly describe that all analyses were intention-to-treat ("All analyses were intention-to-treat and used the maximum available data, resulting in variation in sample size per test").

17a) For each primary and secondary outcome, results for each group, and the estimated effect size and its precision (such as 95% confidence interval)

Does your paper address CONSORT subitem 17a? *

Copy and paste relevant sections from the manuscript (include quotes in quotation marks "like this" to indicate direct quotes from your manuscript), or elaborate on this item by providing additional information not in the ms, or briefly explain why the item is not applicable/relevant for your study

Not applicable for primary analyses, for secondary analyses these results can be found in Table 4.

17a-i) Presentation of process outcomes such as metrics of use and intensity of use

In addition to primary/secondary (clinical) outcomes, the presentation of process outcomes such as metrics of use and intensity of use (dose, exposure) and their operational definitions is critical. This does not only refer to metrics of attrition (13-b) (often a binary variable), but also to more continuous exposure metrics such as “average session length”. These must be accompanied by a technical description how a metric like a “session” is defined (e.g., timeout after idle time) [1] (report under item 6a).

	1	2	3	4	5	
subitem not at all important	○	○	○	○	○	essential

Does your paper address subitem 17a-i?

Copy and paste relevant sections from the manuscript (include quotes in quotation marks "like this" to indicate direct quotes from your manuscript), or elaborate on this item by providing additional information not in the ms, or briefly explain why the item is not applicable/relevant for your study

Metrics of engagement are clearly described in the Results section ("On average, parents completed close to two-thirds of the EMI pre-survey prompts and one-third of the EMI post-survey prompts, with approximately two-thirds completing at least half of all prompts. Pre-EMI survey response rates were the following: 36 participants completed 0-24% of prompts; 29 completed 25-49%; 47 completed 50-74%; 70 completed 75-100%; and 2 exceeded 100%, indicating multiple completions of the same prompt. Post-EMI survey response rates were lower, with 108 participants completing 0–24%, 31 completing 25–49%, 24 completing 50–74%, 18 completing 75–100%, and 3 exceeding 100%." as well as in Figure 2 and Figure 3).

17b) For binary outcomes, presentation of both absolute and relative effect sizes is recommended

Does your paper address CONSORT subitem 17b? *

Copy and paste relevant sections from the manuscript (include quotes in quotation marks "like this" to indicate direct quotes from your manuscript), or elaborate on this item by providing additional information not in the ms, or briefly explain why the item is not applicable/relevant for your study

We did not assess binary outcomes, so this is not applicable.

18) Results of any other analyses performed, including subgroup analyses and adjusted analyses, distinguishing pre-specified from exploratory

Does your paper address CONSORT subitem 18? *

Copy and paste relevant sections from the manuscript (include quotes in quotation marks "like this" to indicate direct quotes from your manuscript), or elaborate on this item by providing additional information not in the ms, or briefly explain why the item is not applicable/relevant for your study

We assessed associations between demographic characteristics and EMI completion rates ("Pearson pair-wise associations were conducted to examine associations between pre-EMI completion rates and parent demographic characteristics...").

18-i) Subgroup analysis of comparing only users

A subgroup analysis of comparing only users is not uncommon in ehealth trials, but if done, it must be stressed that this is a self-selected sample and no longer an unbiased sample from a randomized trial (see 16-iii).

	1	2	3	4	5	
subitem not at all important	○	○	○	○	○	essential

Does your paper address subitem 18-i?

Copy and paste relevant sections from the manuscript (include quotes in quotation marks "like this" to indicate direct quotes from your manuscript), or elaborate on this item by providing additional information not in the ms, or briefly explain why the item is not applicable/relevant for your study

This was not done, as assessing efficacy was not the focus of this study.

19) All important harms or unintended effects in each group
(for specific guidance see CONSORT for harms)

Does your paper address CONSORT subitem 19? *

Copy and paste relevant sections from the manuscript (include quotes in quotation marks "like this" to indicate direct quotes from your manuscript), or elaborate on this item by providing additional information not in the ms, or briefly explain why the item is not applicable/relevant for your study

No harms or unintended effects were observed.

19-i) Include privacy breaches, technical problems

Include privacy breaches, technical problems. This does not only include physical “harm” to participants, but also incidents such as perceived or real privacy breaches [1], technical problems, and other unexpected/unintended incidents. “Unintended effects” also includes unintended positive effects [2].

	1	2	3	4	5	
subitem not at all important	○	○	○	○	○	essential

Does your paper address subitem 19-i?

Copy and paste relevant sections from the manuscript (include quotes in quotation marks "like this" to indicate direct quotes from your manuscript), or elaborate on this item by providing additional information not in the ms, or briefly explain why the item is not applicable/relevant for your study

No privacy breaches occured. However, participants did report technical problems - this is reported in the Methods section ("Due to a technical issue, approximately half of participants in the pilot encountered a problem with survey delivery...").

19-ii) Include qualitative feedback from participants or observations from staff/researchers

Include qualitative feedback from participants or observations from staff/researchers, if available, on strengths and shortcomings of the application, especially if they point to unintended/unexpected effects or uses. This includes (if available) reasons for why people did or did not use the application as intended by the developers.

	1	2	3	4	5	
subitem not at all important	○	○	○	○	○	essential

Does your paper address subitem 19-ii?

Copy and paste relevant sections from the manuscript (include quotes in quotation marks "like this" to indicate direct quotes from your manuscript), or elaborate on this item by providing additional information not in the ms, or briefly explain why the item is not applicable/relevant for your study

We state that participants reported encountering technical issues via the user satisfaction questionnaire ("In line with our technical issues reported above, one-third of participants reported encountering technical issues while using the app, such as not receiving some surveys").

DISCUSSION

22) Interpretation consistent with results, balancing benefits and harms, and considering other relevant evidence

NPT: In addition, take into account the choice of the comparator, lack of or partial blinding, and unequal expertise of care providers or centers in each group

22-i) Restate study questions and summarize the answers suggested by the data, starting with primary outcomes and process outcomes (use)

Restate study questions and summarize the answers suggested by the data, starting with primary outcomes and process outcomes (use).

	1	2	3	4	5	
subitem not at all important	○	○	○	○	○	essential

Does your paper address subitem 22-i? *

Copy and paste relevant sections from the manuscript (include quotes in quotation marks "like this" to indicate direct quotes from your manuscript), or elaborate on this item by providing additional information not in the ms, or briefly explain why the item is not applicable/relevant for your study

Study questions and results are summarized at the beginning of the Discussion ("This pilot randomised controlled trial evaluated the feasibility, engagement, and acceptability of a small-scale prototype of the Daily Growth parenting program... The study met its recruitment target and demonstrated a high retention rate. Engagement with pre-EMI surveys exceeded expectations, although post-EMI survey completion was lower than the target. Parents generally responded positively to the program, finding the resources useful. However, certain demographic groups were underrepresented").

22-ii) Highlight unanswered new questions, suggest future research

Highlight unanswered new questions, suggest future research.

	1	2	3	4	5	
subitem not at all important	○	○	○	○	○	essential

Does your paper address subitem 22-ii?

Copy and paste relevant sections from the manuscript (include quotes in quotation marks "like this" to indicate direct quotes from your manuscript), or elaborate on this item by providing additional information not in the ms, or briefly explain why the item is not applicable/relevant for your study

Suggestions for future research are presented towards the end of the Discussion ("Findings suggest several directions for future research and program refinement. Investigating how parent gender influences program efficacy is important, as mothers and fathers may differ in their parenting approaches[78-80], indicating potential for further tailoring. Additionally, optimizing the timing of intervention delivery is crucial. While in-the-moment interventions can be effective[81,82], some parents may be more receptive to learning new skills outside moments of high emotional dysregulation[82]. Further research should explore the best timing and delivery strategies to maximize impact").

20) Trial limitations, addressing sources of potential bias, imprecision, and, if relevant, multiplicity of analyses

20-i) Typical limitations in ehealth trials

Typical limitations in ehealth trials: Participants in ehealth trials are rarely blinded. Ehealth trials often look at a multiplicity of outcomes, increasing risk for a Type I error. Discuss biases due to non-use of the intervention/usability issues, biases through informed consent procedures, unexpected events.

	1	2	3	4	5	
subitem not at all important	○	○	○	○	○	essential

Does your paper address subitem 20-i? *

Copy and paste relevant sections from the manuscript (include quotes in quotation marks "like this" to indicate direct quotes from your manuscript), or elaborate on this item by providing additional information not in the ms, or briefly explain why the item is not applicable/relevant for your study

Limitations of the study are described ("However, the pilot was limited to testing a 2-week prototype version of Daily Growth, whereas the program is intended to run for 6 weeks. While the short-term feasibility of EMI was demonstrated, it remains uncertain whether parents will maintain engagement with twice-daily prompts over an extended period.
The pilot study had other limitations. The sample, while adequate for a pilot, was relatively small and not fully representative of the broader parent and caregiver population, limiting the generalizability of findings. Additionally, technical issues with post-EMI survey delivery affected data collection for some participants, highlighting the need for improvements in platform functionality") - however, due to this study focusing on feasibility rather than efficacy, we did not describe limitations relating to potential bias in the preliminary efficacy outcomes.

21) Generalisability (external validity, applicability) of the trial findings

NPT: External validity of the trial findings according to the intervention, comparators, patients, and care providers or centers involved in the trial

21-i) Generalizability to other populations

Generalizability to other populations: In particular, discuss generalizability to a general Internet population, outside of a RCT setting, and general patient population, including applicability of the study results for other organizations

	1	2	3	4	5	
subitem not at all important	○	○	○	○	○	essential

Does your paper address subitem 21-i?

Copy and paste relevant sections from the manuscript (include quotes in quotation marks "like this" to indicate direct quotes from your manuscript), or elaborate on this item by providing additional information not in the ms, or briefly explain why the item is not applicable/relevant for your study

We discuss that generalizability is limited, as this was a pilot trial including a small sample ("The sample, while adequate for a pilot, was relatively small and not fully representative of the broader parent and caregiver population, limiting the generalizability of findings").

21-ii) Discuss if there were elements in the RCT that would be different in a routine application setting

Discuss if there were elements in the RCT that would be different in a routine application setting (e.g., prompts/reminders, more human involvement, training sessions or other co-interventions) and what impact the omission of these elements could have on use, adoption, or outcomes if the intervention is applied outside of a RCT setting.

	1	2	3	4	5	
subitem not at all important	○	○	○	○	○	essential

Does your paper address subitem 21-ii?

Copy and paste relevant sections from the manuscript (include quotes in quotation marks "like this" to indicate direct quotes from your manuscript), or elaborate on this item by providing additional information not in the ms, or briefly explain why the item is not applicable/relevant for your study

The program format has since been changed to an app-based format, which may make the program less prone to technical issues ("Future iterations should prioritize improving the technical infrastructure and consider a mobile app format to streamline user experience and minimize disruptions"). Timing and delivery strategies may also be optimized in a future trial. However, the general format of the program (using prompts, no human involvement) will likely not be changed significantly.

OTHER INFORMATION

23) Registration number and name of trial registry

Does your paper address CONSORT subitem 23? *

Copy and paste relevant sections from the manuscript (include quotes in quotation marks "like this" to indicate direct quotes from your manuscript), or elaborate on this item by providing additional information not in the ms, or briefly explain why the item is not applicable/relevant for your study

Retrospective Trial Registration (Australian New Zealand Clinical Trials Registry): ACTRN12624001410549

24) Where the full trial protocol can be accessed, if available

Does your paper address CONSORT subitem 24? *

Cite a Multimedia Appendix, other reference, or copy and paste relevant sections from the manuscript (include quotes in quotation marks "like this" to indicate direct quotes from your manuscript), or elaborate on this item by providing additional information not in the ms, or briefly explain why the item is not applicable/relevant for your study

A full trial protocol is not available.

25) Sources of funding and other support (such as supply of drugs), role of funders

Does your paper address CONSORT subitem 25? *

Copy and paste relevant sections from the manuscript (include quotes in quotation marks "like this" to indicate direct quotes from your manuscript), or elaborate on this item by providing additional information not in the ms, or briefly explain why the item is not applicable/relevant for your study

Information about funding can be found in the Acknowledgements section ("The current study was funded by an internal Deakin University Faculty of Health Research Capacity Building Grant Scheme (HAtCH) grant").

X27) Conflicts of Interest (not a CONSORT item)

X27-i) State the relation of the study team towards the system being evaluated

In addition to the usual declaration of interests (financial or otherwise), also state the relation of the study team towards the system being evaluated, i.e., state if the authors/evaluators are distinct from or identical with the developers/sponsors of the intervention.

	1	2	3	4	5	
subitem not at all important	○	○	○	○	○	essential

Does your paper address subitem X27-i?

Copy and paste relevant sections from the manuscript (include quotes in quotation marks "like this" to indicate direct quotes from your manuscript), or elaborate on this item by providing additional information not in the ms, or briefly explain why the item is not applicable/relevant for your study

Conflicts of interest are described at the end of the manuscript ("None declared. We note that the authors/evaluators are identical to the developers/sponsors of the intervention").

About the CONSORT EHEALTH checklist

As a result of using this checklist, did you make changes in your manuscript? *

- ○ yes, major changes
- ◉ yes, minor changes
- ○ no

What were the most important changes you made as a result of using this checklist?

Added more details in the Methods, for instance regarding randomization.

How much time did you spend on going through the checklist INCLUDING making changes in your manuscript *

I spent roughly 15 hours.

As a result of using this checklist, do you think your manuscript has improved? *

- (x) yes
- ( ) no
- ( ) Sonstiges:

Would you like to become involved in the CONSORT EHEALTH group?

This would involve for example becoming involved in participating in a workshop and writing an "Explanation and Elaboration" document

- ( ) yes
- (x) no
- ( ) Sonstiges:

Auswahl löschen

Any other comments or questions on CONSORT EHEALTH

Meine Antwort

STOP - Save this form as PDF before you click submit

To generate a record that you filled in this form, we recommend to generate a PDF of this page (on a Mac, simply select "print" and then select "print as PDF") before you submit it.

When you submit your (revised) paper to JMIR, please upload the PDF as supplementary file.

Don't worry if some text in the textboxes is cut off, as we still have the complete information in our database. Thank you!

Final step: Click submit !

Click submit so we have your answers in our database!

Senden

Alle Eingaben löschen

Geben Sie niemals Passwörter über Google Formulare weiter.

Dieser Inhalt wurde nicht von Google erstellt und wird von Google auch nicht unterstützt. - Nutzungsbedingungen - Datenschutzerklärung

Sieht dieses Formular verdächtig aus? Bericht

Google Formulare
